# Defect Engineering in Large‐Scale CVD‐Grown Hexagonal Boron Nitride: Formation, Spectroscopy, and Spin Relaxation Dynamics

**DOI:** 10.1002/smll.202506874

**Published:** 2025-10-31

**Authors:** Ivan V. Vlassiouk, Yueh‐Chun Wu, Alexander Puretzky, Liangbo Liang, John Lasseter, Bogdan Dryzhakov, Ian Gallagher, Sujoy Ghosh, Nickolay Lavrik, Ondrej Dyck, Andrew R. Lupini, Marti Checa, Liam Collins, Harry M. Meyer, Huan Zhao, Farzana Likhi, Kai Xiao, Ilia Ivanov, David Glasgow, Alexander Tselev, Benjamin Lawrie, Sergei Smirnov, Steven J. Randolph

**Affiliations:** ^1^ Center for Nanophase Materials Sciences Oak Ridge National Laboratory Oak Ridge TN 37831 USA; ^2^ Materials Science and Technology Division Oak Ridge National Laboratory Oak Ridge TN 37831 USA; ^3^ Chemical Sciences Division Oak Ridge National Laboratory Oak Ridge TN 37831 USA; ^4^ Materials Science and Engineering University of Houston Houston TX 77004 USA; ^5^ CICECO ‐ Aveiro Institute of Materials and Department of Physics University of Aveiro Aveiro 3810 Portugal; ^6^ Department of Chemistry and Biochemistry New Mexico State University Las Cruces NM 88003 USA

**Keywords:** bombardment, CVD, defects, hBN, ODMR, photoluminescence, Raman

## Abstract

Recently, numerous techniques have been reported for generating optically active defects in exfoliated hexagonal boron nitride (hBN), which hold transformative potential for quantum photonic devices. However, achieving on‐demand generation of desirable defect types in scalable hBN films remains a significant challenge. Here, it is demonstrated that formation of negative boron vacancy defects, *V*_B_^−^, in suspended, large‐area CVD‐grown hBN is strongly dependent on the type of bombarding particles (ions, neutrons, and electrons) and irradiation conditions. In contrast to suspended hBN, defect formation in substrate‐supported hBN is more complex due to the uncontrollable generation of secondary particles from the substrate, and the outcome strongly depends on the thickness of the hBN. Different defect types are identified by correlating spectroscopic and optically detected magnetic resonance features, distinguishing boron vacancies (formed by light ions and neutrons and emitting at 800 nm) from other optically active defects emitting at 650 nm assigned to anti‐site nitrogen vacancy (*N*_B_ *V*_N_) and reveal the presence of additional “dark” paramagnetic defects that influence spin‐lattice relaxation time (*T*_1_) and zero‐field splitting parameters, all of which strongly depend on the defect density. These results underscore the potential for precisely engineered defect formation in large‐scale CVD‐grown hBN, paving the way for the scalable fabrication of quantum photonic devices.

## Graphical abstract

The controlled formation of defects in large‐area CVD‐grown hBN using various irradiation methods is demonstrated. Defect type and properties depend on particle type (He^+^, Ne^+^, Ar^+^, neutrons, and electrons), substrate effects, and film thickness. Spectroscopy and ODMR distinguish between defect types, enabling scalable quantum photonic device fabrication through engineered defect control.

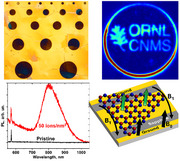

## 1 Introduction

Optically active defects in various solid‐state materials that can be treated as two‐level quantum systems have become a versatile platform for developing emerging quantum technologies.^[^ ^1^, ^2^ ^]^ The recent surge in the exploration of such defects in 2D materials has been driven by their unique properties and, in part, by a wide variety of such van der Waals materials that can enable a plethora of functionalities.^[^ ^3^ ^]^ It is anticipated that such versatile 2D heterostructures should allow the development of integrated quantum photonic systems tuned to specific applications. Such defects have been studied in many 2D materials primarily focusing on transition metal chalcogenides (TMDs)^[^ ^4^ ^]^ and hexagonal boron nitride (hBN).^[^ ^5^, ^6^ ^]^ The latter recently received particular attention due to its wide‐bandgap (6 eV) where defects exhibiting single‐photon emitter (SPE) properties in a wide range of emission energies have been demonstrated.^[^ ^7^, ^8^, ^9^ ^]^

Defects in hBN with broadband emission near 800 nm were recently assigned to negatively charged boron vacancy (*V*_B_^−^), which has a spin‐triplet ground state,^[^ ^10^ ^]^ while the origin of 650 nm emission is still debated with some reports assigning it to anti‐site nitrogen vacancy (*N*_B_ *V*_N_),^[^ ^11^ ^]^ or carbon containing defects.^[^ ^12^ ^]^ The first report on paramagnetic defects created in pyrolytic boron nitride by neutron bombardment is over three decades old,^[^ ^13^ ^]^ but only recently it has regained attention.^[^ ^10^, ^14^, ^15^, ^16^, ^17^ ^]^ Such *V*_B_^−^ defects exhibit spin‐dependent optical transitions and have long relaxation times, making them attractive candidates for developing quantum optical devices in various applications, including quantum sensing and communications.^[^ ^18^, ^19^ ^]^ It was shown that boron vacancies can be formed not only by neutron bombardment but also by electrons and ions of different types.^[^ ^20^ ^]^

Most of the previously reported experiments on defect introduction were performed on either high‐quality bulk hBN crystals or on exfoliated sub‐millimeter‐sized samples.^[^ ^3^ ^]^ This is a result of the need to start with defect‐free samples, and technologically relevant CVD (chemical vapor deposition)‐grown hBN films have lacked that quality and scale. Recently, we^[^ ^21^ ^]^ and others^[^ ^22^, ^23^, ^24^ ^]^ have demonstrated a facile but robust method of high‐quality hBN film deposition on a large‐scale using molecular nitrogen and various solid boron sources; it allowed achieving an extremely low density of defects comparable with that of bulk hBN crystals. Thickness control of hBN films from monolayer to >100 nm was demonstrated by adjusting catalyst composition.^[^ ^21^ ^]^ This allows for the synthesis of high quality hBN films suitable for the rational design of defects with desired properties.

Here, we investigate the formation of optically active defects in large‐scale CVD grown hBN films using different types of particle bombardment (ions, neutrons, and electrons) and demonstrate the selective formation of specific defect types. We focus on the 800 nm emission (preferentially formed by light ions and neutrons) attributed to *V*_B_^−^ defects, that competes with defects of different origins emitting near 650 nm (mostly induced by heavy ions) assigned to anti‐site nitrogen vacancy (*N*_B_ *V*_N_). The thickness of hBN plays a critical role in defect formation, and the outcomes differ between suspended hBN films and those on substrates. This distinction arises from poor control over the type and energy/spatial distributions of secondary particles generated upon collision with the underlying substrate that leads to the formation of mixed defects. We use cathodoluminescence (CL), photoluminescence (PL), Raman spectroscopy, Kelvin probe microscopy, and optically detected magnetic resonance (ODMR) for deeper characterization and understanding of the formation mechanisms of these defects. Using ODMR, we confirm *V*_B_^−^ ground triplet state, and we observe a strong correlation between the spin relaxation times (*T*_1_) and the paramagnetic defect densities that influence *T*_1_ through a cross‐relaxation mechanism. Our findings highlight the importance of balancing photoluminescence brightness and *T*_1_, both of which strongly depend on the defect density. *T*_1_ depends on the environment: at room temperature it decreases from 15 µs at low defect density with weak dependence on the external magnetic field to less than 1 µs at high defect density and strong dependence on the field.

The general conclusion is that in high quality CVD‐grown hBN films, *V*_B_^−^ defects, among others, can be controllably produced yielding a significant potential for large‐scale quantum photonic devices. Nevertheless, reproducible device fabrication and optimized performance on a large scale would ultimately require fine tuning of synthesis to achieve a uniform hBN thickness on the wafer scale.

## 2 Results and Discussion

The penetration depth for a particle into hBN depends on multiple factors including the particle mass, kinetic energy, and angle of incidence, all of which need to be taken into account when generating the defects. If the incident particles penetrate through the hBN layer, they will interact with the substrate, scatter, and possibly backscatter or generate other particles. It introduces complexity into defect generation mechanisms and their spatial localization. For example, bombardment of Si/SiO_2_ ‐ commonly used as substrates in many hBN experiments ‐ by He⁺ ions leads to strong photoluminescence at ≈700 nm, which sometimes mistakenly is attributed to hBN (Figure S1, Supporting Information). The He^+^ beam at 30 keV used in this study has a penetration depth or end‐of‐range (EOR) length of ≈240 nm.^[^ ^15^ ^]^ Importantly, due to the synthesis conditions, most of the hBN films used in this study are thinner than 250 nm (Figures S2–S5, Supporting Information). Thus, to decouple the effects of the substrate during the bombardment, we compare defect formation in suspended hBN membranes and hBN films on bulk substrates.

### 2.1 Suspended hBN Membranes

**Figure 1:**
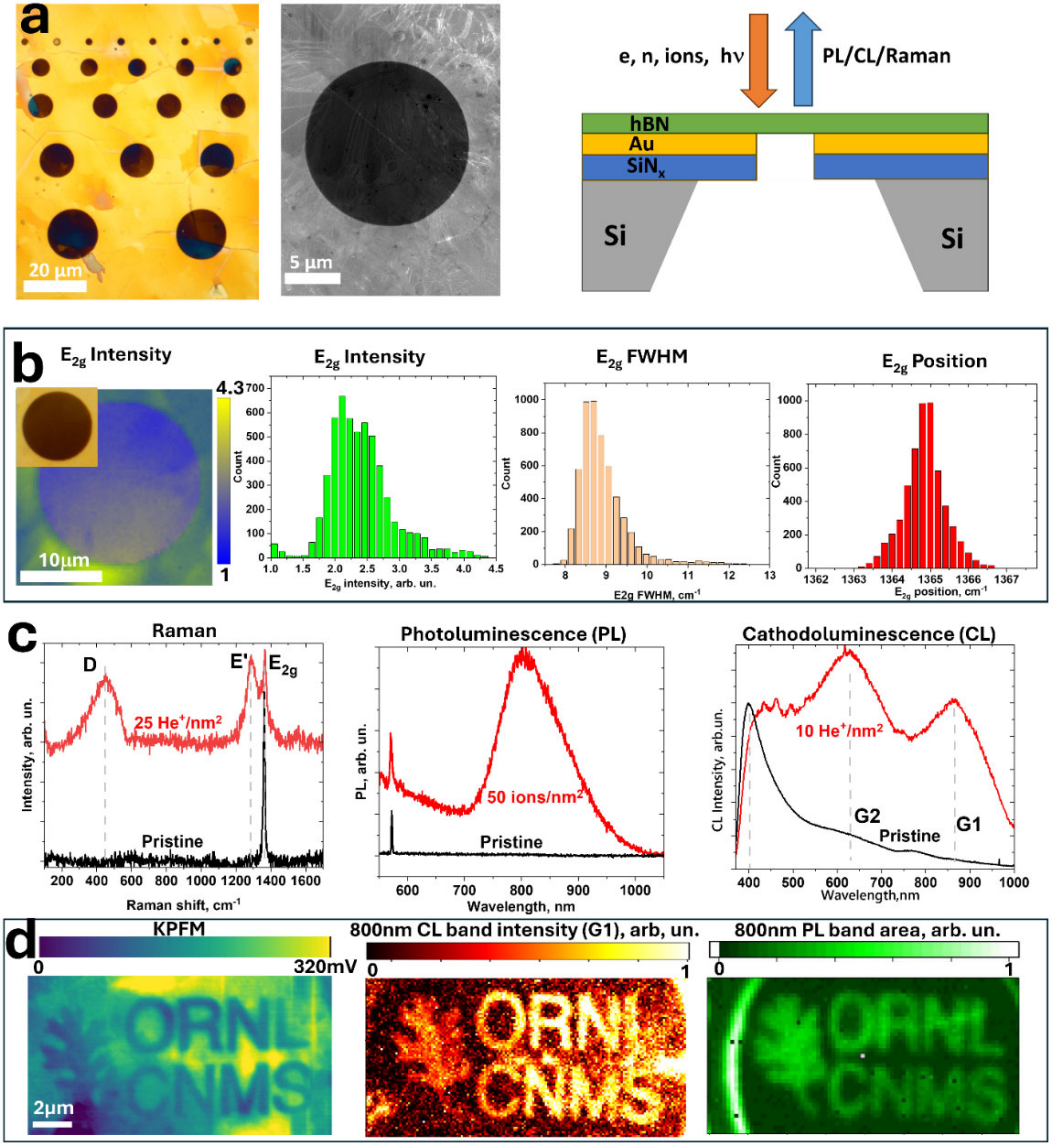
Description of suspended hBN samples and the main spectroscopic features induced by He^+^ bombardment. a) Optical microscope image of suspended hBN on a Si/SiN_x_/Au membrane (left). SEM image of the same sample (center) and a schematic of the chip (right). b) Characteristic Raman maps of suspended CVD‐grown hBN, along with histograms of *E*_2g_ peak intensity, FWHM, and position. c) Changes in Raman spectra (left), photoluminescence (PL) (center), and cathodoluminescence (CL) (right) after bombardment by 30 keV He^+^ ions. The ion doses are indicated in the figure. d) Maps of suspended, patterned hBN membranes with L ≈120 nm thickness obtained by KPFM (left), CL (center), and PL (right).

As shown in **Figure** **1**a, CVD‐grown hBN films are transferred onto Si/SiN_x_/Au membranes and thus have regions that are suspended and on top of gold. The gold layer was deposited on top of SiN_x_ to screen the strong 700 nm SiN_x_ fluorescence that makes the spectroscopic characterization of hBN very hard (Figure S6, Supporting Information) and simulate the appearance of hBN on a microwave (MW) waveguide used later in the ODMR study. The optical microscope image highlights different colors arising from the thickness nonuniformity of the hBN film. Most of the hBN is 90–120 nm thick and has a goldish color, while the islands are ≈240 nm thick and have a pinkish color. The thickness of hBN was determined by optical reflectance spectra and confirmed by (destructive) plasma‐focused ion beam (PFIB) etching coupled with atomic force microscopy (AFM) measurements (Figures S2–S5, Supporting Information). For hBN suspended over large holes, the film′s color also varies due to the thickness variation, which can be monitored by reflectance or transmittance spectra. These nonuniformities in the thickness can be taken into account in data analysis, as explained later.

A typical Raman map of suspended CVD hBN is shown in Figure 1b along with the histogram of the *E*_2g_ band over the whole measured area. The *E*_2g_ band corresponds to the in‐plane vibration of sp^2^ hBN at 1365 cm^−1^ and is considered a primary metric of the hBN quality and uniformity. The map reveals weaker Raman intensity for suspended hBN, at least for ≈120 nm thick samples. This can be rationalized by an increased reflectance from the gold. At the excitation wavelength (532 nm), the reflection from gold in air is ≈0.7, but it is different at the interface with the hBN film and is strongly depends on hBN thickness (Figure S2, Supporting Information). Our typical hBN samples on gold show approximately twice the Raman *E*_2g_ intensity compared with suspended regions (Figure S7, Supporting Information), while some samples of other thicknesses can show an increase as much as fivefold possibly due to plasmon excitations in Au. The FWHM of the *E*_2g_ band in the histogram has a mean value of ≈8.5 cm^−1^, only slightly larger than the 6–8 cm^−1^ FWHM for typical exfoliated samples.

After bombardment of hBN with ions, two additional Raman bands appear; we denote them *E’*, at ≈1290 cm^−1^, and *D*, at 450 cm^−1^. Figure 1c shows the case of irradiation with 30 keV He^+^ ions. The main *E*_2g_ Raman line also significantly broadens with increased defect density. While *E’* was previously reported for exfoliated samples after irradiation,^[^ ^15^ ^]^ the *D* band was identified only for bulk crystals.^[^ ^25^ ^]^ Both these bands can also be observed in the anti‐Stokes part of the Raman signal (Figure S8, Supporting Information). The negatively charged *V*_B_^−^ defect has *D*_3h_ symmetry and is predicted to have ≈1300 and ≈325 cm^−1^ vibration modes^[^ ^26^ ^]^ that closely correlate with the observed positions of the *E’* and *D* lines. Therefore, we attribute these two modes to *V*_B_^−^. Additional evidence supporting the assignment of these bands to *V*_B_^−^ is presented in the following sections.

The introduction of defects is accompanied by the appearance of strong photoluminescence (PL) with a maximum at 800–900 nm (Figure 1c, center) and cathodoluminescence (CL). In the latter, an additional 650 nm emission peak is also observed; we label these bands G1 and G2, respectively (Figure 1c, right). A sharp feature at ≈575 nm corresponds to the Raman signal from hBN. As mentioned above, there is no PL on most areas in hBN prior to irradiation with only rare SPE‐like hotspots indicating a very high quality of our “as synthesized” hBN (Figure S9, Supporting Information). Similarly, in CL “as synthesized” hBN has much weaker emission in the UV–vis spectrum due to defect‐related bands,^[^ ^27^ ^]^ but they are likely formed during electron bombardment^[^ ^28^, ^29^, ^30^, ^31^, ^32^ ^]^ as supported by the results presented in the next sections. Stronger emission further in the UV has been reported in the literature^[^ ^33^ ^]^ but due to our CL detector the range here is limited to operation at 350–1000 nm.

Focused ion beams can pattern such defects in a desired pattern. Figure 1d shows images of the area patterned with a 25 He^+^ nm^−2^ dose recorded by three different techniques: Kelvin probe force microscopy (KPFM), CL, and PL. Importantly, AFM topography does not resolve the patterned region, while KPFM clearly contrasts the bombarded regions. We note that KPFM contrast reflects the net electrostatic environment and may include contributions from trapped charge, band bending, and surface adsorbates; therefore, it is charge‐sensitive but not defect‐specific. Lower measured potentials on the irradiated areas likely arise from the embedded He⁺ ions and newly formed charged defects and their counterions.

**Figure 2:**
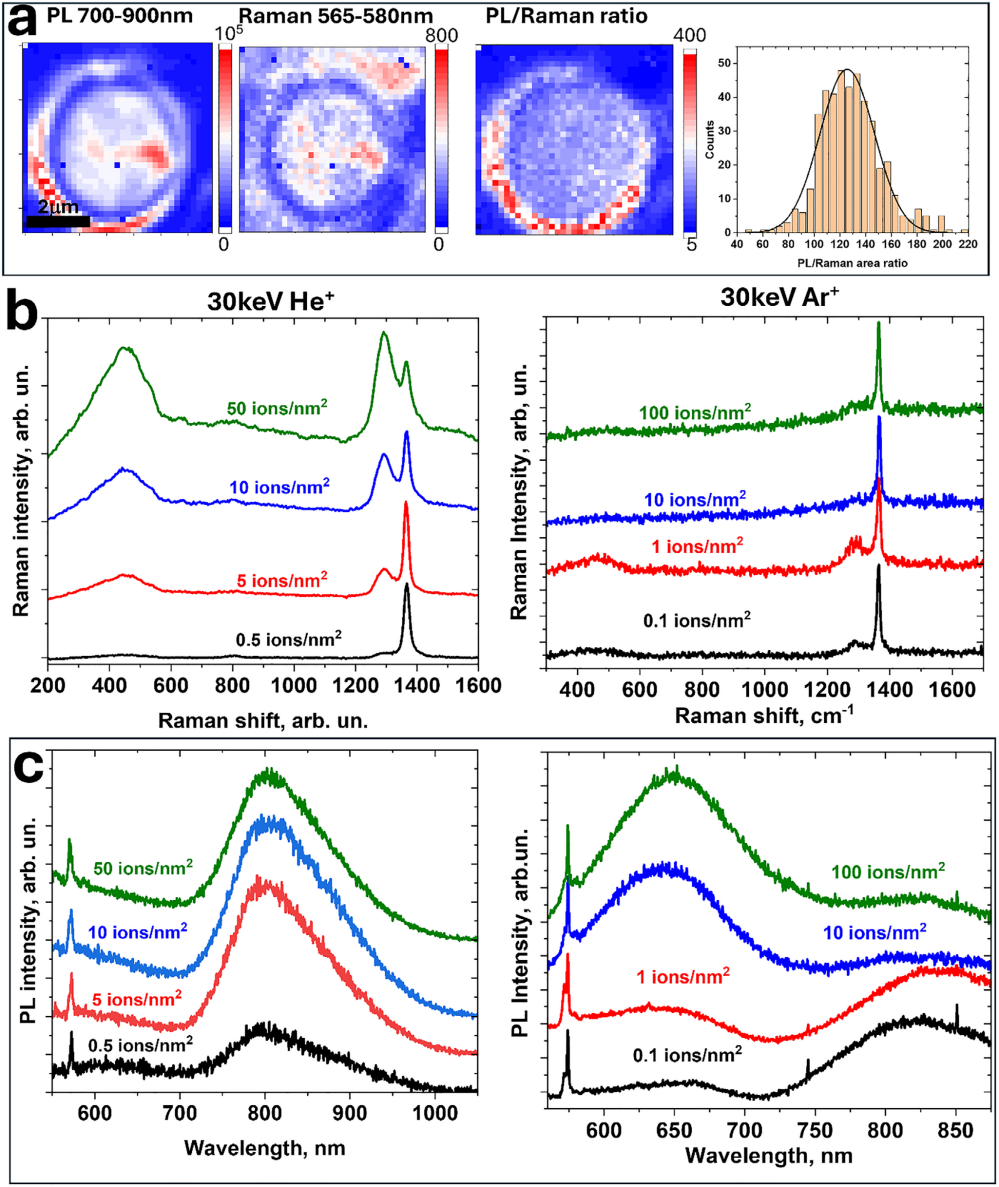
PL signal @800 nm normalized to hBN thickness and spectroscopic variations in suspended hBN under bombardment by various ions and dosages. a) Maps of PL @800 nm (left) and Raman signal for He^+^ irradiated suspended sample for the 50 ions nm^−2^ dosage, along with the PL/Raman ratio and its histogram over the suspended region (right) for. b) Raman spectra of suspended hBN for He^+^ (left) and Ar^+^ (right) 30 keV bombardment. c) Same as (b), but for PL spectra.

**Figure** **2**a shows PL and Raman maps of a suspended hBN membrane bombarded with 50 He^+^ nm^−2^ dose at 30 keV. This is the only energy used here for He^+^ ions. The maps show integrated intensities over the two spectral ranges, 700–900 nm (PL) and 565–580 nm, the latter of which corresponds to the combined *E′* and *E*_2g_ Raman signals. Note a strong fluorescence background signal from the exposed circular SiN_x_ membrane edge uncovered by gold (Figure S6, Supporting Information). Regions with high/low intensities coincide for PL and Raman maps, suggesting that it is due to the hBN thickness variation ‐ thicker hBN regions produce stronger PL and Raman. The PL/Raman ratio map gives a constant value, independent of hBN thickness, indicating that the structure/density of the defects has only a weak dependence on the kinetic energy of He^+^ ions as they traverse the entire hBN thickness and dissipate their energy. Indeed, the observed ratio evens out the original maps′ nonuniformities and demonstrates a narrow ratio distribution (Figure 2a, right). Thus, PL normalization by the Raman intensity provides a reasonable metric for estimating *V*_B_^−^ yield under different bombardment parameters for the thicknesses below EOR.

**Figure 3:**
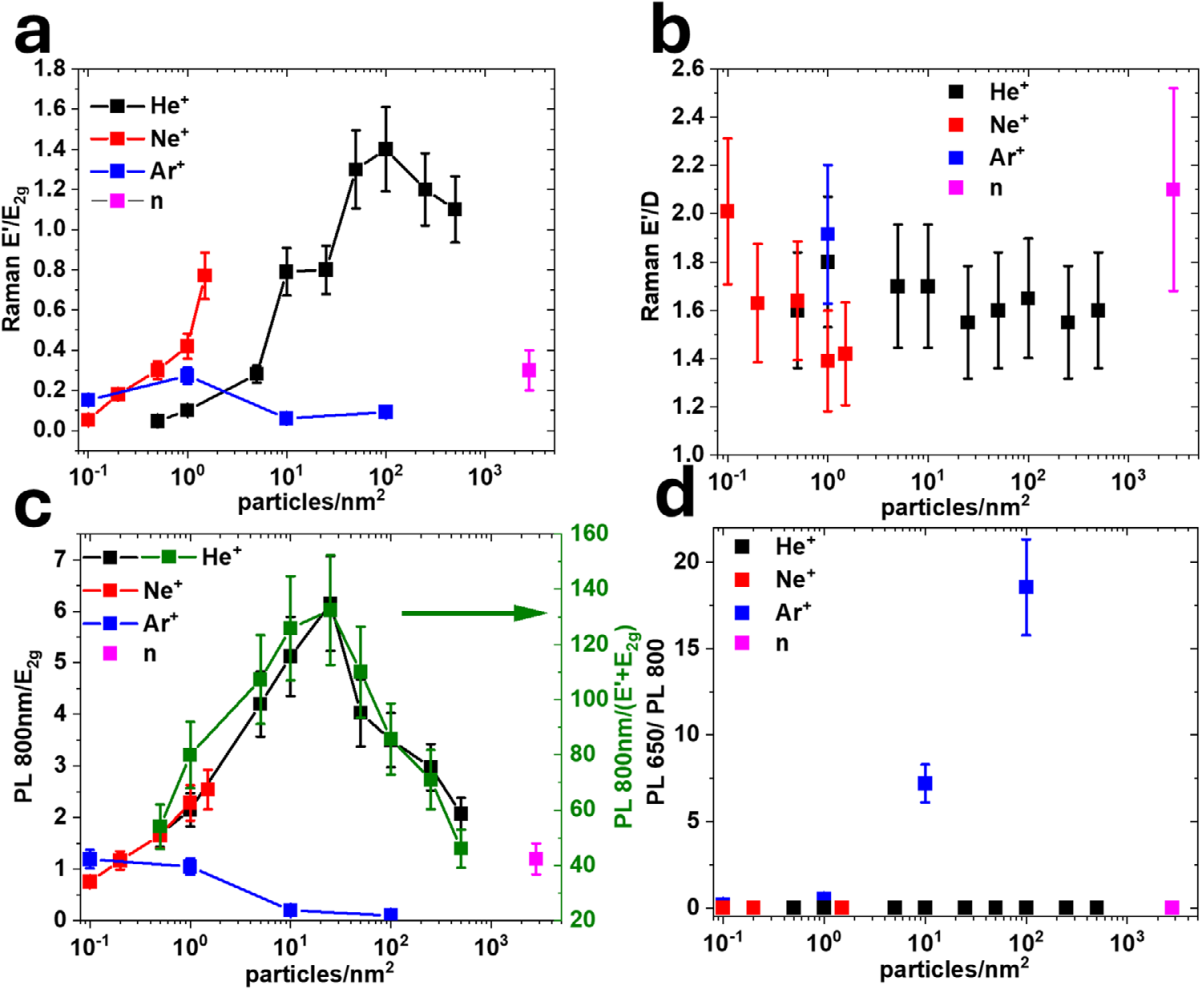
Spectroscopic assessment of suspended hBN under bombardment by different ions, He^+^ (black), Ne^+^ (red), and Ar^+^ (blue), and neutrons (magenta). a) Intensity ratio of Raman bands E’ to *E*_2g_. b) Intensity ratio of Raman bands E’/D. c) PL intensity at 800 nm normalized by *E*_2g_ Raman signal; area ratios for He⁺ are highlighted in green. d) Intensity ratio of PL bands at 800 and 650 nm.

Figure 2b,c compares changes in the Raman and PL spectra for He^+^ and Ar^+^ bombardment (left and right, respectively) using the same 30 keV ion energies. Two obvious differences can be recognized. First of all, lighter He^+^ ions induce larger *D* and *E’* bands in the Raman spectra, while for heavier Ar^+^ ions, these peaks have much lower intensities and are present only at low dosages. Second, the PL shows a correlated behavior with Raman – the 800 nm peak is observed for He^+^ at all dosages but only at low dosages for Ar^+^, for which at large dosages another, 650 nm band overwhelms the 800 nm band. **Figure** **3** summarizes these observations with finer details.

Heavy ions have more efficient energy transfer to boron and nitrogen and thus are more effective in forming complex defects with knocked out atoms. Such a trend was previously observed for graphene.^[^ ^34^ ^]^ Figure 3a illustrates how the defects, evaluated as the *E′/E*_2g_ intensity ratio, evolve as a function of dosages for ions of different mass: He^⁺^ (black), Ne^⁺^ (red), Ar^⁺^ (blue), and neutrons (magenta). For the lightest ion He^+^, much higher doses are required for the *E″* feature to appear, which eventually reaches a maximum at ≈ 100 ions nm^−2^ and declines. The heavier Ne^+^ ions produce defects at lower dosages. The heaviest Ar^+^ ions succeed in making such defects at even lower dosages ‐ the dosage of a maximum for *E′/E*_2g_ ratio for Ar^+^ is roughly two orders of magnitude lower than for He^+^. At the same time, the maximum *E′* intensity for Ar^+^ irradiated hBN is a substantially lower compared to Ne^+^ and especially He^+^. These results indicate that the *E′* band corresponds to defects produced with greater yield under lighter ion bombardment.

The trend agrees with the expected decline of the energy transfer efficiency for particles of small masses but further lowering the mass of irradiating particles (by moving to neutron or electron irradiation, for example) is counterproductive. A declining cross‐section for energy transfer not only requires much higher dosages for defect production but also leads to longer EOR and thus a greater effect of interaction with the substrate for thin hBN films. Neutrons as irradiating particles of suspended hBN are closer in performance to light ions even though our source has a broad energy distribution (Figure S13, Supporting Information) and there is a route for a thermal neutron transmutation reaction with ^10^B leading to the formation of high energy ^7^Li, ^4^He, and gamma photons (Section S5, Supporting Information). The Raman and PL spectra of suspended hBN after neutron bombardment exhibit the characteristic *D* and *E′* Raman modes along with the 800 nm PL band.

The results of keV electron bombardment are more complex. Large electron dosages (≈10^6^ e nm^−2^) are required to observe 650 nm PL (with weak 800 nm shoulder) and 1580 cm^−1^ Raman on “as‐synthesized” suspended hBN, however, possible electron‐beam‐induced carbon deposition^[^ ^35^ ^]^ complicates the analysis (Section S6, Supporting Information). It is not apparent why electrons are so prone to producing such defects. We suspect that it is due to a significant difference in the cross sections for producing initial defects and their “alteration” by second collision, where the second one is significantly greater. It is apparent from Figure S14c (Supporting Information) that no recognizable single atom knockout defects are visible under electron irradiation; all defects appear with multiple atoms missing, and the number and size of those defects grow with the dosage. Measurements of cross‐sections for inelastic scattering of electrons do show some indications for a preference of lower energy excitation that may lead to an increase in the defect formation efficiency.^[^ ^36^, ^37^ ^]^

Notably, the *E′/D* ratio remains nearly constant (1.68 ± 0.26 for 532 nm excitation), regardless of whether neutrons or different ion species are used and regardless of the dose chosen for defect creation (Figure 3b). This strongly suggests that the *D* band is either a different vibrational mode of the same defect, as was predicted for *V*_B_^−^,^[^ ^26^ ^]^ or another defect type that incidentally always forms in the same proportion, which is unlikely. This stands in stark contrast to graphitic materials, where different types of defects also produce two Raman bands identified as a *D* peak at ≈1330 cm^−1^ and *D′* at ≈1620 cm^−1^ along with their combination peak, *D+D′*, at ≈2940 cm^−1^. The irradiation‐dependent changes in the ratio of intensities of those peaks, *D/D′*, have allowed for the characterization of different defect types.^[^ ^38^ ^]^

As described above, variations in PL intensity at 800 nm for He^+^ irradiated samples follows the thickness and, when normalized by the *E*_2g_ intensity, it gives a uniform image (Figure 2a). Similar normalization can be assessed for samples irradiated with other ions, as shown in Figure 3c. First, normalization by either *E*_2g_ (black) or *E′+E*_2g_ integrated intensity (green) gives a similar dependence. Second, the 800 nm PL intensity mirrors the trends of *E′* and *D* (Figure 3a,b) across different doses and ion types, suggesting that the same defects are responsible for both the Raman and 800 nm PL features.

Figure 3d shows the PL intensity ratio between 650 and 800 nm bands for samples irradiated with different ions. For suspended hBN membranes, a pronounced 650 nm peak appears in the PL spectra only of the Ar^+^ bombarded samples (Figures 2c and 3c), at least for the doses used. Nevertheless, in the CL spectra it can be observed for He^+^ bombarded samples too; its intensity increases and the peak position hypsochromically shifts with increasing dose (Figure S14, Supporting Information). Notably, this peak is also observed in the PL of He^+^ bombarded samples taken after CL experiments. (Figure S10, Supporting Information), which suggests that its appearance is caused by irradiation with electrons. The 650 nm PL and a 1580 cm^−1^ Raman band can be attributed to the formation of N_B_V_N_ defects^[^ ^11^ ^]^ ‐ an anti‐site nitrogen vacancy in which a nitrogen atom migrates to a boron vacancy, leaving behind a neighboring nitrogen vacancy, i.e., suggesting that e‐beam bombardment leads to the transformation of *V*_B_^−^ (prepared by He^+^ bombardment) into *N*_B_ *V*_N_.

**Figure 4:**
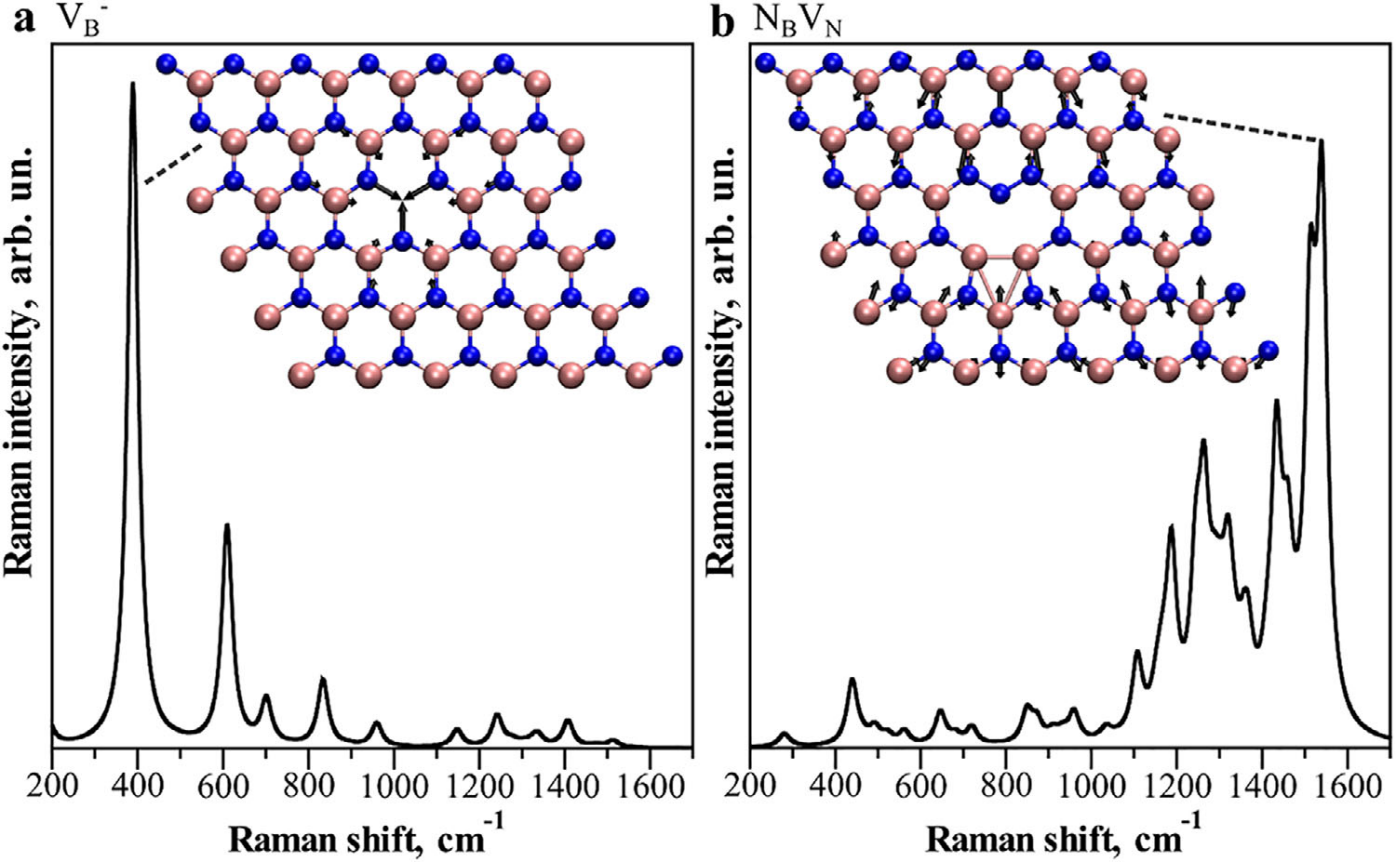
Calculated Raman spectra of hBN with different defects. a) *V*_B_^−^ defect and b) *N*_B_ *V*_N_ defect. The atomic vibration patterns of dominant Raman peaks are shown as insets. Black arrows indicate the direction and amplitude of the atomic vibrations. B atoms are in orange color and N atoms in blue color.

To understand the experimental defect‐related Raman spectra, we carried out first‐principles density functional theory (DFT) calculations of monolayer hBN with a *V*_B_^−^ defect and a *N*_B_ *V*_N_ defect, as shown in **Figure** **4**. Raman spectra are found to be strongly dependent on the defect structure. In Figure 4a, a Raman peak at ≈389 cm^−1^ is dominant in the spectrum (for lower defect density it shifts to 460 cm^−1^, see Experimental section), which corresponds to a characteristic phonon mode closely related to the *V*_B_^−^ defect as its vibrations are largely localized around the vacancy site. This is consistent with a prior work predicting such a defect phonon mode at ≈325 cm^−1^.^[^ ^26^ ^]^ In contrast, for the *N*_B_ *V*_N_ defect (Figure 4b), the strongest Raman peak is around 1540 cm^−1^ with a vibration pattern more or less distributed across the whole structure. Such defect‐dependent Raman features corroborate the experimental observations that the *V*_B_^−^ defect yields a strong Raman defect peak around 450 cm^−1^, while the *N*_B_ *V*_N_ defect leads to a strong Raman band around 1580 cm^−1^. Our calculations slightly underestimate the frequencies of Raman peaks, but this does not affect the conclusion. The presence of 1580 cm^−1^ on gold and absence on the suspended samples (Figure 2b) we attribute to Raman signal plasmonic enhancement by gold, as also seen for *E*_2g_ peak as well.

We attempted to resolve the 650 nm PL hBN defect structure using STEM in a few‐layer hBN sample that served as a model for our multilayer hBN samples. In monolayer hBN, the distinct contrast between N and B atoms makes it possible to resolve the atomic structure of the defects with practically unambiguous element assignment.^[^ ^39^ ^]^ However, in a multilayer AA’ stacking, where B and N atoms are on top of each other, it is next to impossible to distinguish between even the simplest defects, such as *V*_B_ and *V*_N_, relying exclusively on the contrast. Moreover, we found it difficult to conclusively determine whether the observed defects by STEM were induced by prior to ion bombardment or generated under the electron beam during imaging. In fact, multiple complex defects are formed even during bilayer hBN imaging, and pre‐existing defects ‐ possibly introduced by ion beams^[^ ^40^ ^]^ ‐ enlarged under the STEM beam after ≈10^8^ e nm^−2^ dosage (Figure S14, Supporting Information).

### 2.2 hBN on Substrates

As previously mentioned, the types and yields of defects in hBN on a substrate can differ from those in suspended samples for the same type of irradiation, especially when the hBN thickness, *L*, is lower than the EOR of the incident particles. When an incident beam fully penetrates the hBN overlayer (*L* < EOR), its interaction with the underlying substrate inevitably introduces additional defects in the overlying hBN. Even when the hBN layer exceeds the ions’ penetration depth (*L* > EOR), a substantial decline of the ion′s kinetic energy by the end of EOR can lead to diverse defect generation mechanisms and variations in the defect yields.

**Figure 5:**
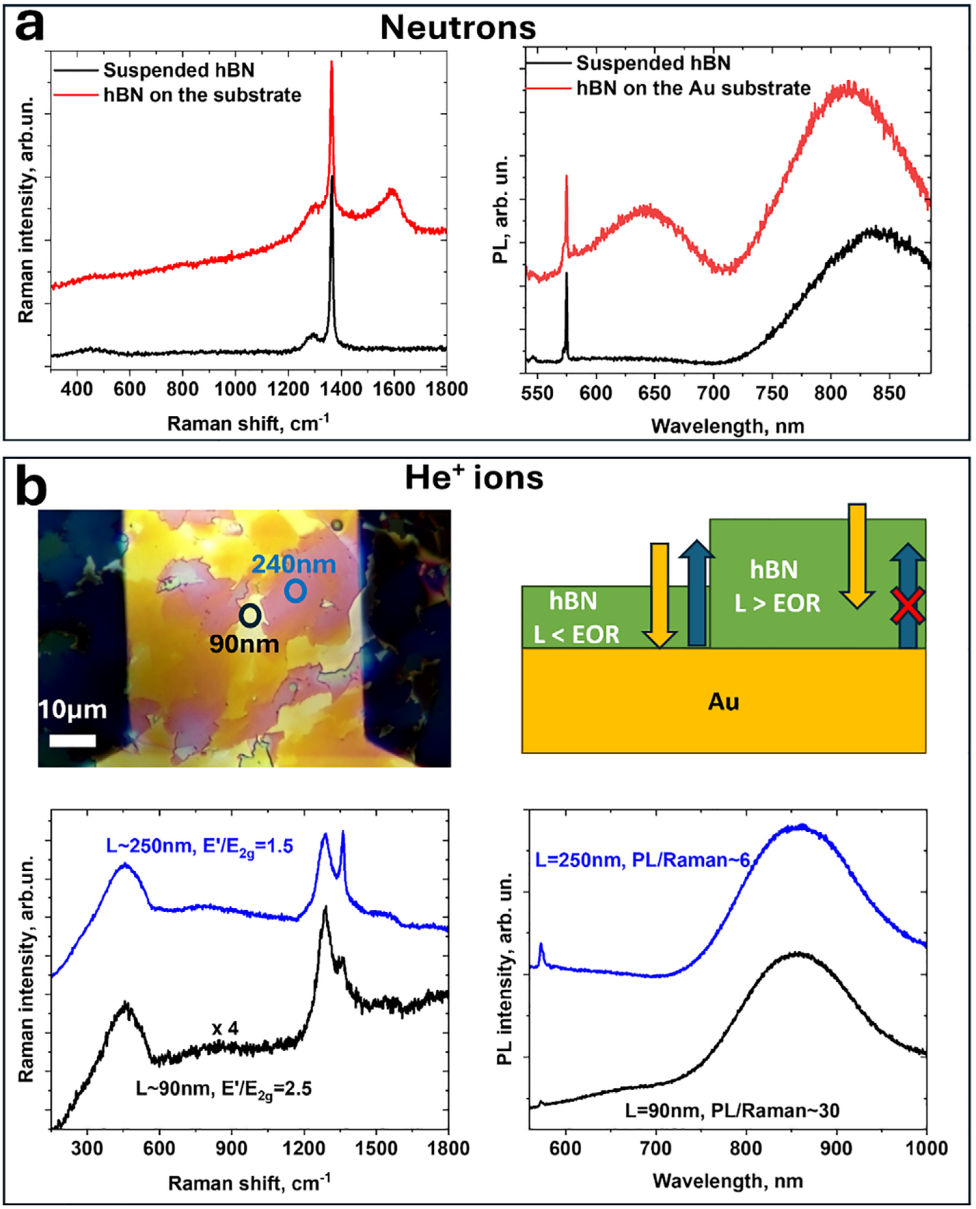
Effects of substrate on defect creation. a) Raman (left) and PL spectra (right) of suspended and supported hBN by the substrate. Note emergence of 1580 cm^−1^ band in Raman and 650 nm PL for supported samples. b) He^+^ ion bombarded hBN on a gold substrate. 50 He^+^ nm^−2^ example. Optical microscope image of a non‐uniform hBN transferred onto a gold substrate with approximate hBN thickness indicated at two points, each marked by a different color (top). Raman and PL spectra corresponding to these two points show characteristics that differ significantly from those observed in suspended samples, emphasizing the importance of considering the effects of substrate (bottom).

Light particles have a longer EOR and thus this effect is more pronounced for them (Section S7, Supporting Information). Secondary particles of the most consequence in this respect are backscattered ions re‐emerging from the substrate in the case of irradiation by He^+^. The effect of the substrate is illustrated in **Figure** **5**. First, we consider neutron irradiation with broad energy distributions (Section S5, Supporting Information) and a very large EOR, allowing the neutron beam to penetrate hBN of all thicknesses used in this study and interact with the Au substrate. The Raman and PL spectra of suspended hBN after neutron bombardment exhibit the characteristic *D* and *E’* Raman modes along with 800 nm PL, similar to the effects observed when hBN is bombarded with light particles, indicating formation of *V*_B_^−^ defects (Figure 5a).

However, the situation changes drastically for supported hBN. A prominent 1580 cm^−1^ Raman band appears, along with pronounced 650 nm PL, previously observed in suspended hBN after e‐beam (Figure S14, Supporting Information) and Ar^+^ (Figure 2) bombardment at high dosages.

Figure 5b shows Raman and PL spectra of hBN on a gold/sapphire substrate that has been irradiated with 50 He^+^ nm^−2^. To highlight how the variations in hBN thickness change the outcome, we intentionally deposited hBN with a significant thickness nonuniformity. The gold stripe serves as a microwave waveguide for ODMR experiments described in the following section. The Raman spectra show that thinner regions (≈90 nm) exhibit a significantly enhanced E’ peak, with *E’/E*_2g_ ratio reaching 2.5. Additionally, the PL signal at 800 nm, normalized by the Raman intensity, reaches values around 30 – significantly greater than those observed in suspended samples (Figure 3a,c). Also note the appearance of a broad shoulder around ≈1580 cm^−1^ that was absent in the suspended samples under similar conditions. He^+^ ions upon reaching gold underneath hBN inelastically scatter and produce secondary electron emission.^[^ ^41^ ^]^ The latter are responsible for the 1580 cm^−1^ shoulder in Raman and 650 nm emission on e‐beam bombarded samples besides the “normally” produced *V*_B_^−^ defects, as for suspended samples.

Interestingly, some samples exhibit hBN “waves”, formed unintentionally during the transfer process, that exhibit a strong 800 nm PL band even with Ar^+^ bombardment, which was never observed for suspended or flat samples on gold substrate (Figure S11, Supporting Information). A similar effect was observed on neutron bombarded samples too. We do not attribute this effect to variations in the effective bombardment angle due to the “waviness” of the hBN film, as no significant dependence was observed when varying the Ar⁺ beam incident angle from 90° to 30°. Nevertheless, it is clear that the greater signal is not only due to the interference contrast either, as a greater density of defects is clearly identifiable by a greater *E’/E*_2g_ for the “wavy” part. Mechanical stress/strain^[^ ^42^, ^43^ ^]^ in “wavy” hBN can be one alternative explanation for the enhanced quantum yield of defects but it does not seem to be sufficient either. This illustrates additional complexity and highlights the importance of substrate effects, which were well controlled in our experiments.

### 2.3 Optically Detected Magnetic Resonance

We will focus on the *V*_B_^−^ defects emitting at 800 nm as the most promising defect type. The assignment of these defects to *V*_B_^−^ has been proposed previously^[^ ^10^ ^]^ and we confirm it here. Besides, we were able to obtain additional important previously unknown information, as described below.

**Figure 6:**
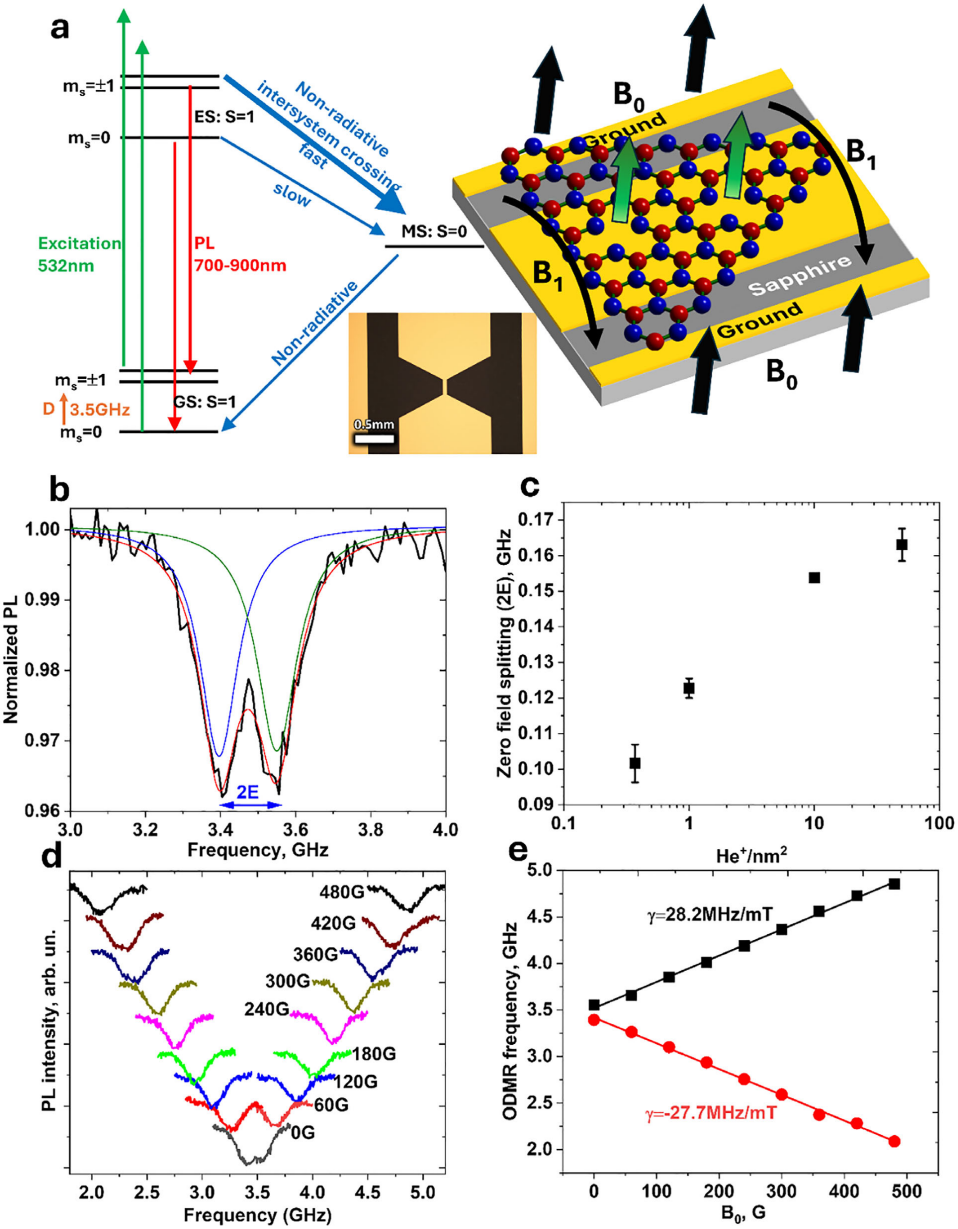
Optically detected magnetic resonance (ODMR) of created defect. a) Simplified energy diagram of the *V*_B_^−^ defect (left) and a schematic of experimental geometry (right), along with an optical microscope image of the coplanar waveguide design (insert). b) Characteristic ODMR spectrum at zero magnetic field (B_0_). c) Dependance of zero‐field splitting (ZFS) parameter *E* on the He^+^ bombardment dosage. The parameter *D* remains unaffected by bombardment d) ODMR spectra at varying B_0_. e) Dependence of ν_1_ and ν_2_ on the B_0_ yielding g≈2.00.

A simplified *V*_B_^−^ energy‐level diagram highlighting major transitions is shown in **Figure** **6**a. The triplet ground state (GS) of the *V*_B_^−^ defect comprises three spin sublevels with *m*_s_ = 0 separated from m_s_ = ±1 by zero‐field splitting (ZFS) characterized by parameter *D*. The excited state (ES) is also a triplet (S = 1) while a metastable state (MS) is a singlet (S = 0).^[^ ^10^, ^16^, ^17^ ^]^ Non‐radiative intersystem crossing (ISC) transitions between S = 1 and S = 0 states are indicated by blue arrows. Excitation from the GS to ES is shown by green arrows, while radiative PL emission from the ES to GS is represented by red arrows. Notably, the ISC transition from ES (S = 1) to MS (S = 0) is faster from m_s_ = ±1 (|±1>) than from m_s_ = 0 (|0>).^[^ ^44^ ^]^ This spin‐dependent ISC rate enables optical detection of spin transitions, a key feature for quantum applications.

For optical detection of magnetic resonance (ODMR) a sample needs to be exposed to a microwave (MW) field, for which gold coplanar waveguides fabricated on sapphire substrates are used. A sample of hBN is placed on such a waveguide and irradiated directly on it. An optical microscope image of a device geometry, along with a schematic of the *V*_B_^−^ defect energy diagram, is shown in Figure 6a. The MW magnetic field (*B*_1_) is oriented along the gold surface, while the applied static magnetic field (B_0_) is perpendicular to the waveguide plane, i.e.*, B*_1_ ⊥ B_o_. Under continuous‐wave (CW) laser excitation, when the MW is off‐resonance, the GS is more spin‐polarized in the |0> state, resulting in maximum PL. However, when the MW field drives transitions to |±1>, the PL intensity decreases due to the enhanced quenching caused by ISC.^[^ ^10^ ^]^ The transitions can be induced by MW excitation not only in the GS but in the ES as well but at different frequency.^[^ ^16^ ^]^

Neglecting hyperfine interactions, the spin Hamiltonian is given by Equation 1, with the Z‐axis oriented perpendicular to the hBN plane:

(1) $$H=D\left(S_{z}^{2}-\frac{S(S+1)}{3}\right)+E\left(S_{x}^{2}-S_{y}^{2}\right)+g\mu_{B}BS$$

Here *E* and *D* are the transverse and axial ZFS parameters aligned with and perpendicular to the symmetry axis of the *V*_B_^−^ center, respectfully. S is the electron spin (S = 1), B_0_ is the external magnetic field, *S*_x,y,z_ are the spin operators, g is the electron Lande g‐factor, and μ_B_ is the Bohr magneton.

Figure 6b shows ODMR spectrum at zero external magnetic field (B_0_ = 0) using CW excitation for a sample after He^+^ irradiation detected at the 800 nm band corresponding to *V*_B_^−^ defects. In the ground state, the splitting between *m*_s_ = 0 and *m*_s_ = ±1 is ν_0_ = *D*/h ≈3.5 GHz. The splitting between *m*_s_ = 1 and *m*_s_ = −1 sublevels is also visible. It is given by E/h at zero field and increases with applied B_0_ as:

(2) $$\nu_{1,2}=\nu_{0}\pm \frac{1}{h}\sqrt{E^{2}+{\left(g\mu_{B}B_{0}\right)}^{2}}$$

Both *D* and *E* are primarily determined by the magnetic dipole–dipole interactions of the spins in the triplet state (GS) and were previously thought to be intrinsic characteristics of the *V*_B_^−^ center.^[^ ^10^ ^]^ We have found that the parameter *E* noticeably depends on the dosage (Figure 6c) while *D* remains unaffected (Figure S12, Supporting Information). Similar behavior was previously observed for NV centers in diamond and hBN flakes.^[^ ^45^, ^46^ ^]^ The value of *E* = 51 MHz at the lowest dose of 0.37 He+ nm^−2^ is close to the previously reported for the ground state of *V*_B_^−[^ ^10^, ^46^ ^]^ but increasing irradiation doses lead to its almost twofold increase at 50 He+ nm^−2^. The increase is quite gradual, close to logarithmic. We found the ODMR contrast to be independent of the He^+^ dose, 3.4 ± 0.7% at room temperature for our bombardment conditions and MW power. To ensure consistency for our ODMR results, we used hBN with a thickness of L≈250 nm for all doses since varying hBN thicknesses can yield different defect densities/types as shown in Figure 5b. We also screened all our ODMR samples to ensure that the 650 nm PL and 1580 cm^−1^ Raman band were absent.

Increasing B_0_ causes the *m*_s_ = ±1 levels to split further apart in accordance with Equation 2, see Figure 6d,e. This Zeeman splitting corresponds to the Lande g‐factor close to that of a free electron, *g*_e_ = 2.00, as was previously reported for *V*_B_^−^ defects.^[^ ^10^ ^]^ The 650 nm PL, however, does not exhibit an ODMR signal, at least within 2.4–5 GHz and at zero field (Figure S17, Supporting Information).

**Figure 7:**
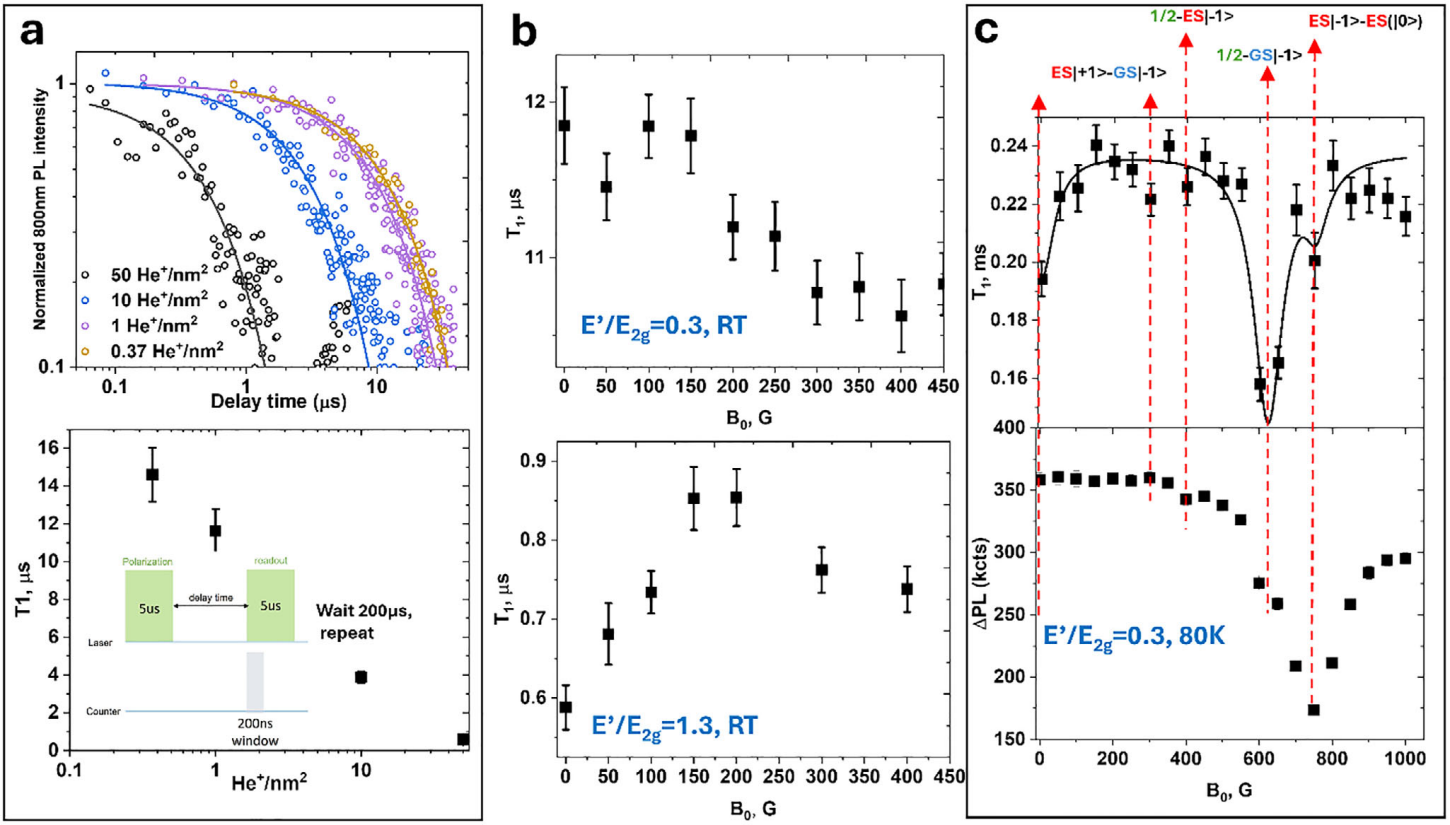
Dependence of the spin‐lattice relaxation time (*T* *_1_*) on the defect density and applied magnetic field. a) Time traces used for *T*_1_ estimation (top) and *T*_1_ time versus He^+^ ion dosages (bottom). b) Room temperature *T*_1_ as a function of the applied magnetic field for low *V*_B_^−^ defect densities (E’/*E*_2g_ = 0.3, top) and high defect densities (E’/*E*_2g_ = 1.3, bottom). c) *T*_1_ as a function of the applied magnetic field for low *V*_B_^−^ defect densities at T = 80 K (top) and ΔPL, a PL decay amplitude (bottom); the time trace was fitted as y0+ΔPL×exp(−t/*T*_1_).

The spin‐lattice relaxation time (*T*_1_) can be measured by a sequence of polarization and delayed readout pulses, as shown in the inset of **Figure** **7**a. The room temperature spin‐lattice relaxation time (*T*_1_) for *V*_B_^−^ defects in exfoliated high‐quality hBN was measured to be around 18 µs,^[^ ^14^ ^]^ which is comparable to 15 µs measured in our CVD‐grown samples at the irradiation dose of 0.37 He^+^ nm^−2^ (*E’/E*_2g_≈0.03). At high defect densities, the reduced distance between defects enhances dipole–dipole interactions between unpaired electron spins facilitating more efficient energy exchange between the spins and the environment.^[^ ^47^ ^]^ This leads to shorter *T*_1_, which decreases to less than 1 µs at the irradiation dose of 50 He^+^ nm^−^^2^ (*E’/E*_2g_ = 1.3)(Figure 7a). The decline has a nearly logarithmic dependence on the dose, similar to that of the *E* dependence. The similarity hints at a possibly common cause. Note that *T*_1_ dependence on the defect density was previously reported for NV centers in diamond at room temperature,^[^ ^45^ ^]^ which switched to a much stronger linear variation of *T*_1_ with the dose at cryogenic temperatures.^[^ ^48^ ^]^ The authors proposed different mechanisms of relaxation ‐ interaction with phonons at high (room) temperature and cross relaxation with neighboring spins at low temperature. We believe that interaction with neighboring spins such as of other defects and restrained He^+^ ions and knockout atoms/ions could be responsible for relaxation in *V*_B_^−^ centers even at room temperature. The same defects cause the changes in *E* as well. Some authors believe that the dependence of *E* and *T*_1_ on ion dosage can be primarily attributed to the increasing crystal damage and even tried to correlate it with the sizes of irradiating ions.^[^ ^46^ ^]^ Although the strains and local electric fields do get introduced by negatively charged boron vacancies,^[^ ^47^, ^49^, ^50^ ^]^ but the similar weak dependence of *E* and *T*_1_ on the bombardment dosages (Figure 3) and dependence of *T*_1_ on magnetic field suggest for significant contribution from magnetic dipole–dipole interaction between *V*_B_^−^ spin and other neighboring spins.

The importance of spin–spin interactions even at room temperature is supported by the weak dependence of *T*_1_ on *B_0_* at low defect densities in both supported and suspended hBN (Figure 7b, top) and the strong dependence observed at high defect concentrations (Figure 7b, bottom).^[^ ^47^ ^]^ Cross‐relaxation mechanisms are more profound at the points of resonance^[^ ^44^, ^51^ ^]^ ‐ where the energy levels of two neighboring spin systems become resonant and induce efficient energy exchange between them. Such points can be observed by varying magnetic field. In the given range up to 400 G, T_1_ at high defect densities (*E’/E*_2g_ = 1.3) initially rises with *B*_o_ but declines beyond 200 G. The level anticrossing in *V*_B_^−^ for the ground state between m_S_ = ‐1 and m_S_ = 0 occurs at B_0_ ≈1250 G but between *m*_S_ = −1 of the ground state and m_S_ = 1 of excited state (for which *D*_e_ ≈2.1 GHz and *E*_e_ ≈74 MHz) the anticrossing occurs near ≈200 G.^[^ ^16^ ^]^ Other dips in PL contrast due to anticrossing with S = 1/2 occur near ≈400 and 600 G.^[^ ^48^ ^]^ A combination of these resonances for *T*_1_ at high fields and the rise away from zero field results in an effective maximum of T_1_ at ≈200 G. The dependence is different at low defect densities (*E’/E*_2g_ ≈0.3), where T_1_ slightly decreases with increasing field.

The anticrossing mechanism, even in samples with low defect density, becomes prominent at low temperatures. Figure 7c (top) presents *T*_1_ as a function of *B*_0_ acquired at T = 80 K, while the ΔPL contrast acquired at the same temperature is shown in Figure 7c (bottom). The levels crossing labels are shown at the top. The most significant decreases in *T*_1_ are observed for ½⇔GS (*m*_s_ = −1) and ES (*m*_s_ = −1)⇔ ES (*m*_s_ = 0). Importantly, we do not observe a significant difference in the peaks between suspended and supported hBN, suggesting similar densities of paramagnetic defects with S = 1/2. However, *T*_1_ for suspended hBN (220 µs) is slightly longer than that of supported hBN (190 µs) at T = 80 K at the same defect densities, which is more than an order of magnitude longer than at RT.

## 3 Conclusion

We report that specific defect types in large‐area CVD‐grown hexagonal boron nitride (hBN) can be selectively formed by choosing the bombarding particles. Among the investigated ions of different masses, neutrons, and electrons, He^+^ ions represent the best choice for preferential creation of negative boron vacancies *V*_B_^−^ for suspended and substrate‐supported hBN with Ne^+^ and neutrons being the second best. These boron vacancies exhibit two characteristic Raman bands at ≈1300 and ≈450 cm^−1^ and emit at 800 nm. Heavier ions (Ar⁺) and electrons within the tested energy range primarily induce other defects with a 1580 cm^−1^ Raman band and 650 nm photoluminescence, which are likely anti‐site nitrogen vacancy (*N*_B_ *V*_N_) defects.

For hBN supported on a substrate, which is more appropriate for many applications, the outcome of irradiation can significantly differ from that of suspended hBN, particularly when the end‐of‐range of the bombarding particles exceeds the hBN thickness. The secondary particles generated upon collisions with the substrate lead to poorly controlled defects even with He^+^ ions, although this choice remains the best overall.

The spin‐lattice relaxation time, *T*_1_, shows a strong correlation with the defect density, decreasing at room temperature from 15 µs at low defect densities to <1 µs at high defect densities following approximately logarithmic dependence. The observed dependence of *T*_1_ on the external magnetic field confirms the role of the cross‐relaxation mechanism and points to the presence of “dark” paramagnetic defects, such as irradiating ions and knocked out atoms/ions. The in‐plane zero‐field splitting parameter, *E*, increases with the defect density close to logarithmically, while the *D* parameter remains unchanged. These findings demonstrate the potential for precise engineering of defect formation in large‐scale CVD‐grown hBN, paving the way for the  fabrication of quantum photonic devices, particularly for quantum sensing and communication applications.

## 4 Experimental Section

### hBN Synthesis and Transfer

A previously reported hBN synthesis approach, utilizing solid boron and molecular nitrogen as precursors, was employed.^[^ ^21^ ^]^ In brief, a 50 µm thick Fe/Ni foil served as a catalytic substrate for growth. 1 g of solid boron precursor was placed in a 2×2″ crucible, with the foil positioned on top. A 3‐inch hot‐wall quartz tube furnace was heated to 1100 °C under a 2.5% Ar/H_2_ mixture. Argon was replaced with nitrogen gas at a flow rate of 1 L min^−1^, and the growth process was continued for 30 min. Subsequently, FeCl_3_ etchant was used to dissolve the foil. The transfer was conducted without a PMMA supporting overlayer, as the hBN film was sufficiently thick to withstand manipulation during the process.

### Defect Introduction

Zeiss Orion NanoFab Helium Ion Microscope was used for Ne^+^ and He^+^, Thermo Scientific Helios 5 Hydra UX DualBeam Multiple Ion Plasma FIB/SEM for Ar^+^, Zeiss Merlin SEM for electron irradiation, and bombardment by neutrons were done at High Flux Isotope Reactor at Oak Ridge National Laboratory (Section S5, Supporting Information). Penetration depth distributions simulations were performed using Stopping Range of Ions in Matter (SRIM) and reported in Section S7 (Supporting Information).

### CL (Cathodoluminescence)

The cathodoluminescence (CL) measurements were conducted using an FEI Quattro environmental SEM equipped with a Delmic Sparc CL collection module, which employs a parabolic mirror to collect CL signals from the sample under electron beam excitation. An electron beam at 5 kV and 110 pA was used with a 700 ms exposure time for CL point spectra acquisition and 150 l mm^−1^ grating was used with a 100 µm input slit to collect those spectra. 2D CL spectral mapping was performed at 5 kV and 230 pA over a 15 × 11 µm area with a pixel density of 140 × 110 using a 200 ms acquisition time per pixel. All CL measurements were conducted at room temperature.

### Raman/PL

The PL and Raman maps were obtained using two different setups, both yielding similar results. The first setup utilized the commercially available InVia Qontor system (Renishaw). Excitation was performed with a 532 nm laser, delivered through a 100× (NA = 0.85) Leica objective. The scattered signal was collected by the same objective and passed through a set of ultra‐narrow notch filters onto 1800 and 300 lines mm^−1^ gratings for Raman and PL measurements, respectively.

The second setup was a custom‐built micro‐PL system. PL excitation was carried out using a 532 nm laser (Excelsior, Spectra Physics, 100 mW) through an upright microscope with a 100x objective (NA = 0.9). The emitted PL light was analyzed by a spectrometer (Spectra Pro 2300i, Acton, f = 0.3 m), which was coupled to the microscope and equipped with 150, 600, and 1800 lines mm^−1^ gratings, as well as a CCD camera (Pixis 256BR, Princeton Instruments).

### Device Fabrication

Regular clean room techniques were used. For Au adhesion, 5 nm Ti / Cr were deposited before Au.

### STEM (Scanning Transmission Electron Microscope)

Atomic resolution high‐angle annular dark field (HAADF) scanning transmission electron microscope (STEM) images of bilayer hBN were acquired using a Nion UltraSTEM 200 operated with an accelerating voltage of 80 kV.

### ODMR (Optically Detected Magnetic Resonance)

For continuous‐wave (CW) optically detected magnetic resonance (ODMR) characterization, the hBN film was transferred onto a pre‐patterned waveguide for microwave (MW) excitation. The MW signal was generated using an OPX+ and Octave system (Quantum Machines), providing a frequency range of 2–18 GHz. An amplifier was used to deliver 26 dBm of MW power to the system. The spin defect ensemble was optically excited using a 516 nm Cobolt laser with a fluence of around 20 W cm^−^^2^. The resulting photoluminescence (PL) was collected by a single‐photon counting module (SPCM, Excelitas) after passing through a 750 nm long‐pass filter. The ODMR contrast was measured by monitoring PL intensity as a function of the swept MW frequency.

### *T*_1_ Relaxation

The *T*_1_ spin relaxation measurement is performed using an all‐optical scheme. The spin state is initially polarized by an optical pulse (516 nm, 5 µs), followed by a second optical pulse (516 nm, 5 µs) for readout. A single photon counting module (SPCM, Excelitas) detects photoluminescence (PL) within a 200 ns window, synchronized with the arrival of the readout pulse. The T_1_ relaxation time is extracted by measuring the PL intensity as a function of the delay between the initialization and readout pulses and fitting the data to a single exponential decay for time constant analysis.

### DFT Calculations

First‐principles DFT calculations were performed using the plane‐wave Vienna Ab initio Simulation Package (VASP).^[^ ^52^ ^]^ The electron–ion interactions were described by the projector‐augmented‐wave (PAW) method. The exchange‐correlation interactions were captured by the local density approximation (LDA).^[^ ^53^ ^]^ For a primitive unit cell of pristine monolayer hBN, atomic positions and lattice constants were optimized until the residual forces were below 0.001 eV Å^−1^ and the cutoff energy was chosen at 500 eV. A gamma‐centered 36×36×1 k‐point sampling was used, and the optimized lattice constants were *a* = *b* = 2.489 Å. A vacuum region of about 27 Å in the *z* direction was used to avoid spurious interactions with the neighboring cells. Then, a 6×6×1 supercell was constructed where a *V*_B_^−^ defect or a *N*_B_ *V*_N_ defect was introduced. The k‐point sampling was reduced to 6×6×1 accordingly. Atomic positions were optimized until the residual forces were below 0.001 eV Å^−1^. Phonon calculations were carried out in this optimized supercell using the finite difference scheme implemented in the Phonopy software.^[^ ^54^ ^]^ Hellmann–Feynman forces in the supercell were computed by VASP for both positive and negative atomic displacements (Δ = 0.01 Å) and then used in Phonopy to construct the dynamic matrix, whose diagonalization provides phonon frequencies and phonon eigenvectors (i.e., vibrations). Raman intensities of the supercell structure were calculated within the Placzek approximation.^[^ ^55^, ^56^ ^]^ Basically, one needs to calculate the derivatives of the dielectric tensors with respect to the atomic displacements for obtaining the Raman tensor. For both positive and negative atomic displacements (Δ = 0.01 Å), the dielectric tensors ε_αβ_ were computed by VASP at the experimental laser frequency 2.33 eV (532 nm) and thus their derivatives were obtained via the finite difference scheme. Based on the phonon frequencies, phonon eigenvectors and the derivatives of dielectric tensors, Raman tensor $\tilde{R}$ and Raman intensity of any phonon mode can be obtained. Finally, a Raman spectrum was obtained after Lorentzian broadening with a full width at half maximum (FWHM) of 30 cm^−1^.

The 6×6 hBN supercell was chosen because calculating both phonon frequencies and Raman intensities for larger supercell structures is computationally demanding. To demonstrate that larger cells can yield more accurate phonon frequencies, a 9×9 supercell with a single *V*_B_^−^ defect was simulated, corresponding to a lower defect concentration. In this case, the characteristic defect Raman mode is blue‐shifted to 460 cm^−1^, in much better agreement with the experimental value of ≈450 cm^−1^. A similar dependence of Raman modes on defect concentration has been reported for monolayer TMDs such as MoS_2_ and MoSe_2_.^[^ ^57^, ^58^ ^]^ In short, the discrepancy between the calculated Raman peak positions in Figure 4 and experimental counterparts likely arises from the higher defect concentration in the smaller supercell calculations, which were chosen due to the high computational cost.

### Statistical Analysis

All bombarded and pristine suspended area mappings had sizes of at least 10 µm^2^, with more than 400 Raman and PL spectra collected from different locations (see, for example, Figures 1b and 2a). Bombarded areas on the waveguides exceeded 200 µm^2^, with a total of more than 1000 spectra acquired (Figure 5b). All spectra were carefully fitted using either Renishaw WIRE or OriginLab software, and the values are reported with standard deviations (see, for example, Figure 3). ODMR spectra and T1 measurements were performed at least at three different locations with the same bombardment dosage (Figures 6 and 7).

## Conflict of Interest

The authors declare no conflict of interest.

## Supporting information



Supporting Information

## Data Availability

The data that support the findings of this study are available in the supplementary material of this article.
