# Supplementary material for: Defect Engineering in Large‐Scale CVD‐Grown Hexagonal Boron Nitride: Formation, Spectroscopy, and Spin Relaxation Dynamics
Source: Small. 2025 Oct 31;22(8):e06874. doi: 10.1002/smll.202506874 (PMC12877981; doi:10.1002/smll.202506874)
Supplement: Supplementary file 1 — Supporting Information [file SMLL-22-e06874-s001.docx]

Supporting Information

Defect Engineering in Large-Scale CVD-Grown Hexagonal Boron Nitride: Formation, Spectroscopy, and Spin Relaxation Dynamics

Ivan V. Vlassiouk*, Yueh-Chun Wu, Alexander Puretzky, Liangbo Liang, John Lasseter, Bogdan Dryzhakov, Ian Gallagher, Sujoy Ghosh, Nickolay Lavrik, Ondrej Dyck, Andrew R. Lupini, Marti Checa, Liam Collins, Harry M. Meyer III, Huan Zhao, Farzana Likhi, Kai Xiao, Ilia Ivanov, David Glasgow, Alexander Tselev, Benjamin Lawrie, Sergei Smirnov and Steven Randolph

1. **Photoluminescence of 300nm SiO_2_/Si bombarded with 30 keV He^+^ beams**…………….………..2

**Figure S1.** *Photoluminescence (PL) of 300 nm SiO₂/Si ..*…………………………………….………2

1. **Determination of hBN thickness using reflectometry, a profilometer, and AFM** ……….………2

**Figure S2.** *Modeling of hBN Film Reflectance on Gold* ……………………………….…………….2

**Figure S3**. *Examples of reflectance spectra of hBN on gold* ………………………….……………..4

**Figure S4.** *Assessment of hBN thickness using AFM* ……………………………….………………..5

**Figure S5.** *Assessment of hBN thickness using profilometer* ……………………….………………..6

1. **Additional Raman and PL measurements** ……………………………………………..…………..7

**Figure S6.** *Fluorescence of the SiNₓ edge uncovered by a gold layer*……………………..………….7

**Figure S7.** *Comparison of hBN E_2g_ Raman band intensity in gold-supported and suspended regions* ………………………………………………………………………………………………..………..8

**Figure S8.** *Stokes and anti-Stokes shifts for the D band for 1.5 ions/nm² Ne⁺ bombardment of suspended hBN* ……………………………………………………………………………...…………9

**Figure S9.** *PL maps of suspended “as-synthesized” hBN* ………………………………...………….10

**Figure S10.** *PL maps of He^+^ bombarded samples taken after CL experiments* …………...………….11

**Figure S11.** *Illustration of the intense 800 nm PL on “wavy” hBN* ………………...………………...12

1. **Dependance of *ν_0,1,2_* on the He^+^ dosage** ………………………………...……………………………13

**Figure S12.** *Dependance of ν_0,1,2_ on the He^+^ dosage* ……………………..…………………………..13

1. **Neutrons bombardment** …………………………………………………….………………………14

**Table S1**. *PT-1 Fluences for three neutron energy ranges* ………………………….………………...14

**Figure S13.** *Neutron (top) and photon (bottom) energy spectra in High Flux Isotope Reactor (HFIR)*………………………………………………………………………………….………………15

1. **Electrons bombardment** ……………………………………………………………….…………….16

**Figure S14.** *Effects of electron beam irradiation on evolution of hBN defects* ………………….……17

1. **End-of-Range (EOR) length estimations by Stopping Range of Ions in Matter (SRIM**)………...17

**Figure S15.** *Simulated, normalized ion penetration depth distributions as a function of ion species and substrate materials*…….…………………………………………………………….…………….17

1. **XPS spectra of hBN after He+ bombardment**

**Figure S16.** *XPS spectra of hBN after He⁺ bombardment* …………………………………………….18

1. **Figure S17.** *ODMR at 650 nm* ………………………………………………………………………19
2. **References** ………………………………………………………………………………….…………19
3. **Photoluminescence of 300nm SiO_2_/Si bombarded with 30 keV He^+^ beams**


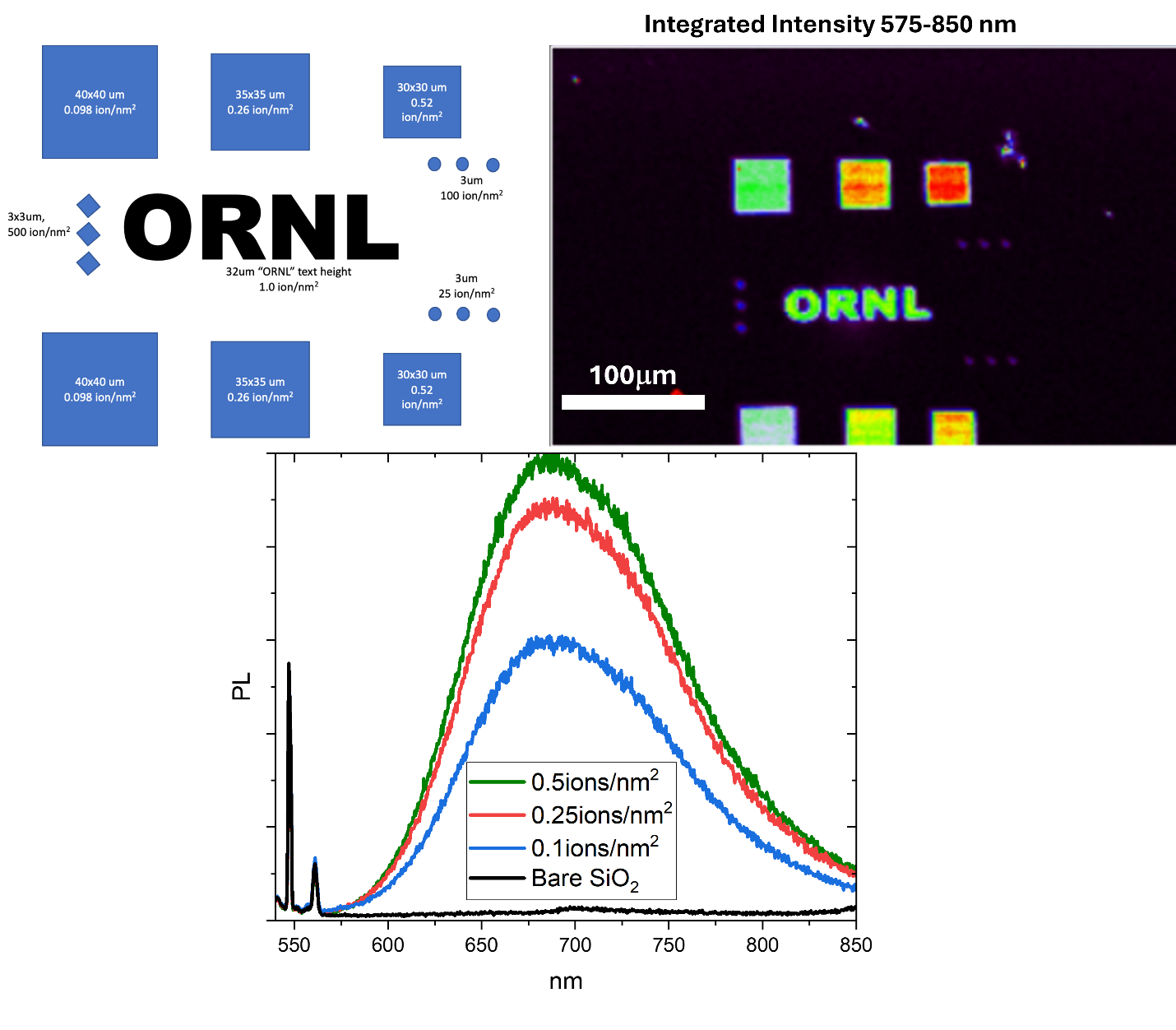


**Figure S1.** *Photoluminescence (PL) of 300 nm SiO₂/Si***.** Bare SiO₂/Si was patterned using 30 keV He⁺ beams with the dosages indicated in the figure. PL maps (integrated intensity from 575–850 nm) reveal a distinct pattern. The PL spectra of bombarded SiO_2_/Si by He^+^ exhibit characteristic Si Raman lines along with a strong, broad emission around 700 nm.

1. **Determination of hBN thickness using reflectometry, a profilometer, and AFM**
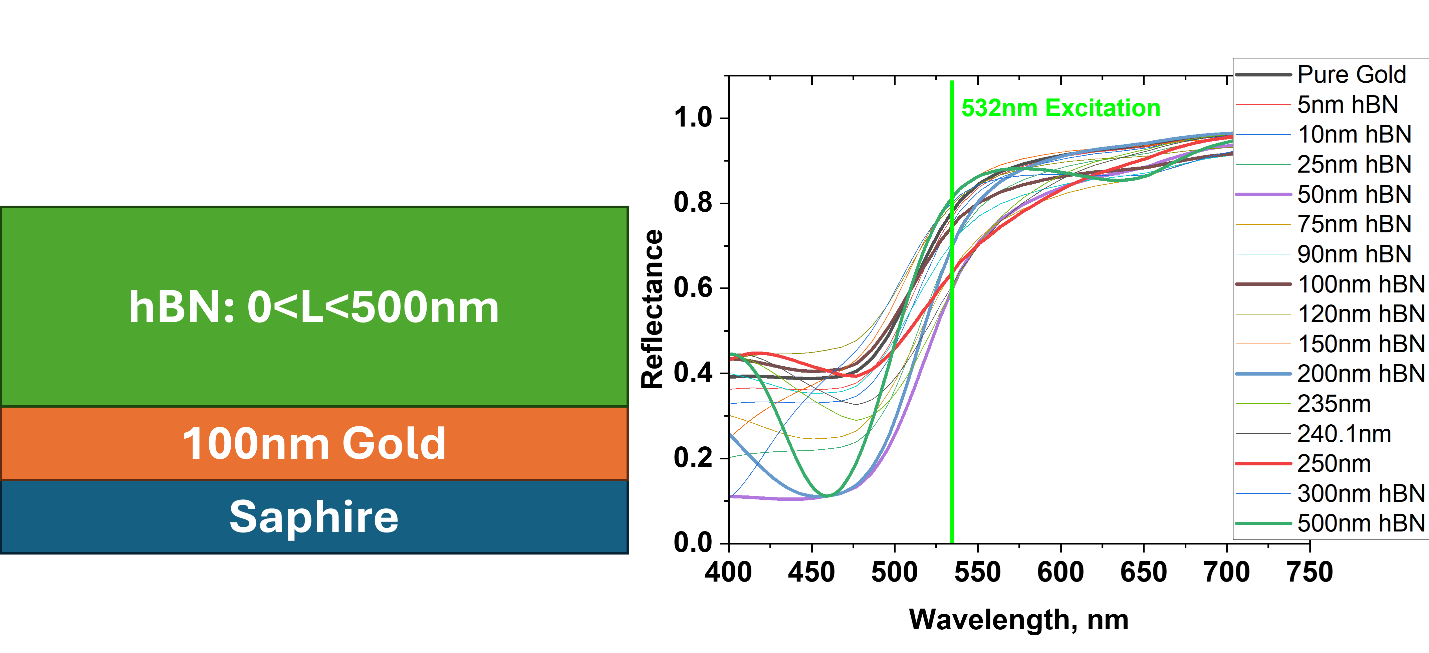


**Figure S2.** *Modeling of hBN Film Reflectance on Gold.*

It is challenging to distinguish between bare gold and gold with a 100 nm hBN overlayer by reflectance spectra. For certain samples, thickness was verified using both AFM and profilometry. The modeling was performed using Filmetrics tool: https://www.filmetrics.com/reflectance-calculator


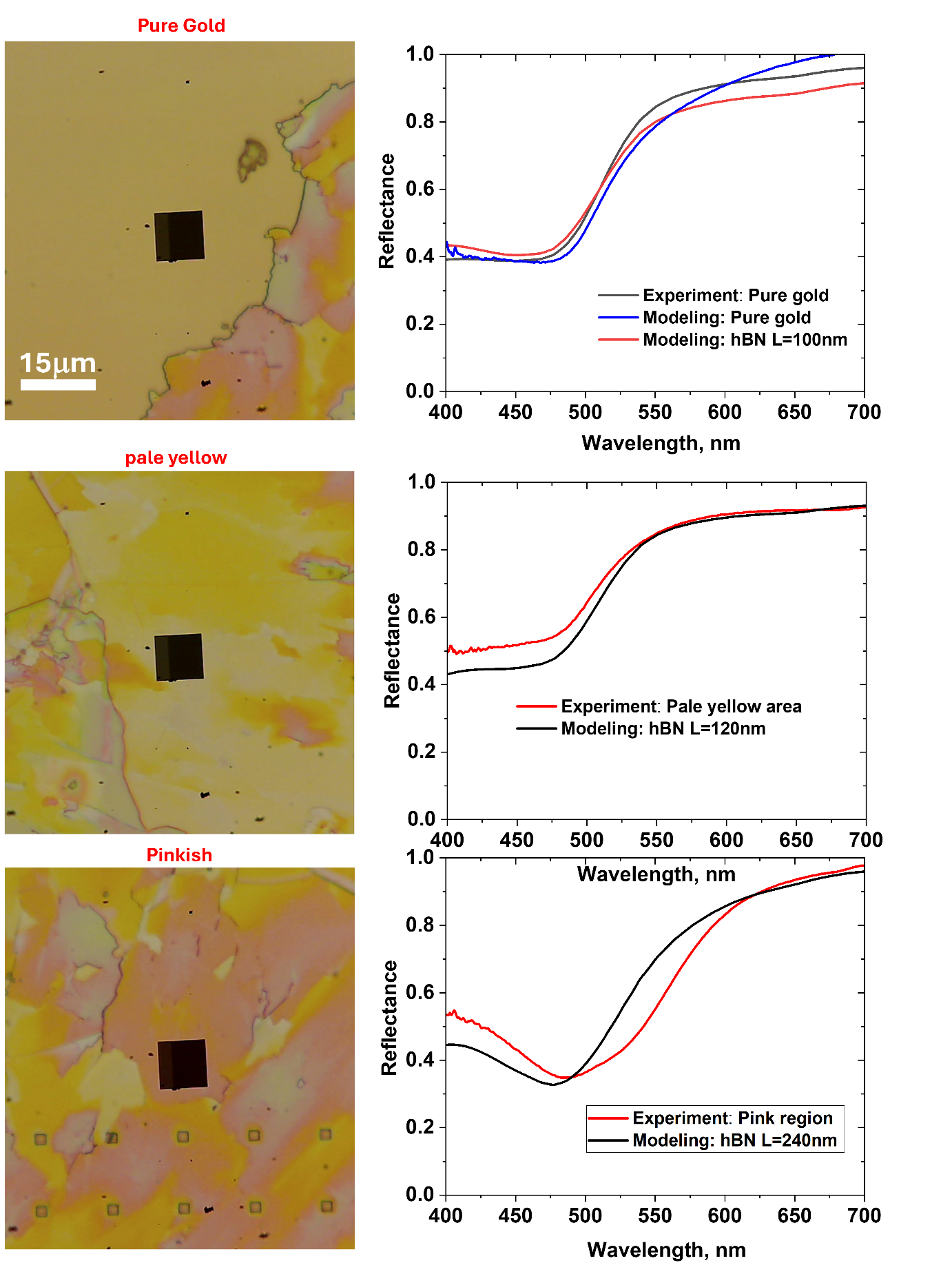


**Figure S3**. *Examples of reflectance spectra of hBN on gold.* The pale yellow color corresponds to an hBN thickness of approximately 120 nm (L ≈ 120 nm), while the pink color corresponds to a thickness of around 240 nm (L ≈ 240 nm)


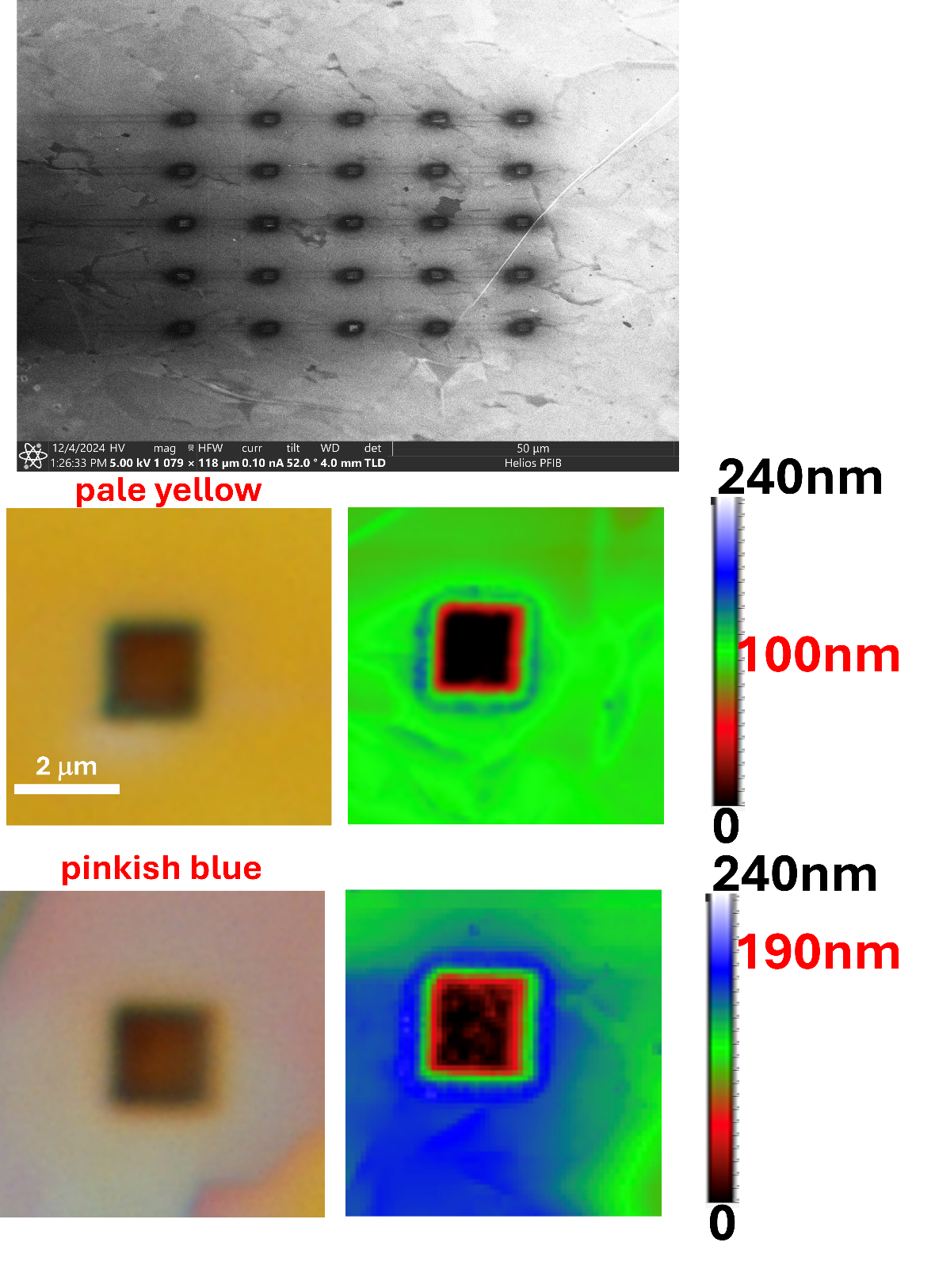


**Figure S4.** *Assessment of hBN thickness using AFM.* 2μm holes were etched in different hBN regions (SEM image, top) using a plasma-focused ion beam (Ar⁺). The completeness of the etching process was determined by an increase in current when the beam etched through nonconductive hBN and reached the gold substrate. AFM was employed to measure the approximate hBN thickness, confirming the estimations based on reflectance data. The pale yellow color corresponds to a thickness of approximately 100 nm (L ≈ 100 nm), while the pinkish-blue color indicates a thickness of around 190 nm (L ≈ 190 nm).


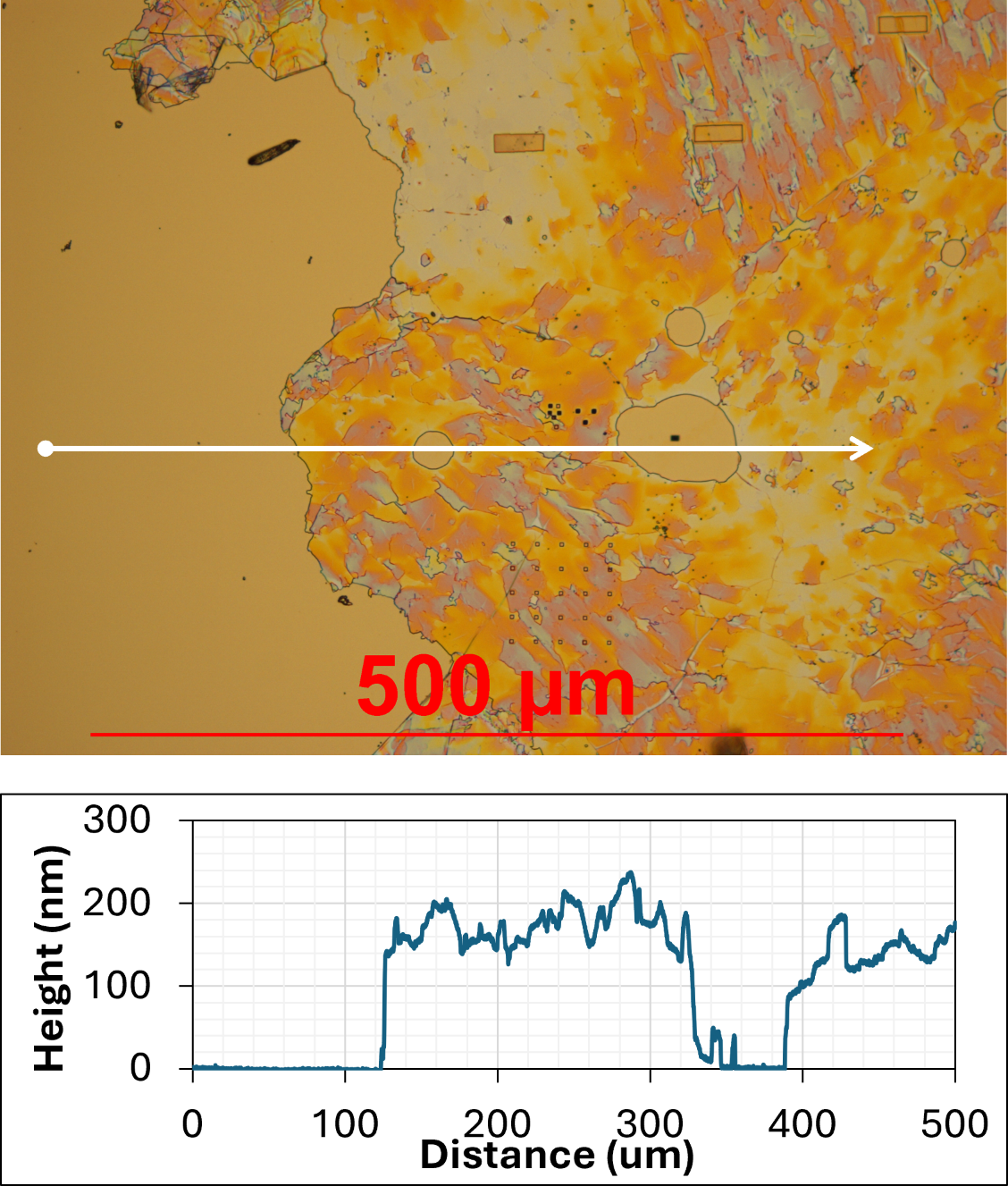


**Figure S5.** *Assessment of hBN thickness using profilometer.* Nonuniform film regions were used to estimate maximum film thickness. Importantly, the film thickness never exceeded 250nm – the penetration depth or End of Range (EOR) for 30 keV He^+^ beams.^1^

1. **Additional Raman and PL measurements.**


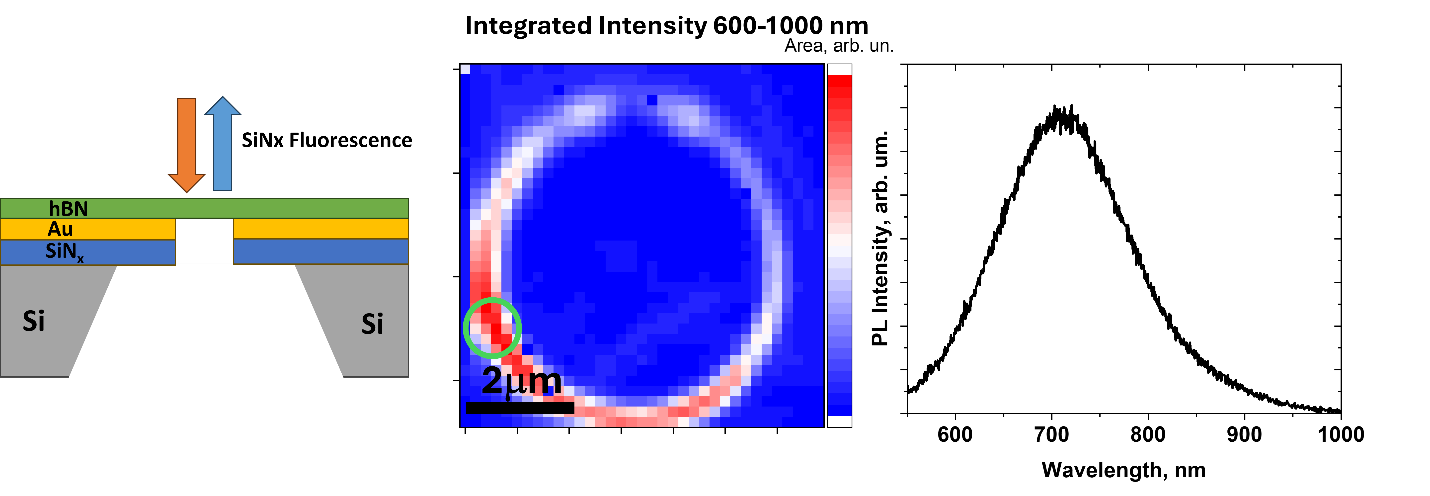


**Figure S6.** *Fluorescence of the SiNₓ edge uncovered by a gold layer.* A sketch of the membrane (left) alongside the integrated fluorescence intensity within the 600–1000 nm range (center). The characteristic fluorescence spectrum of SiNₓ from the edge, highlighted by a green circle on the map, shows strong emission at 700 nm.


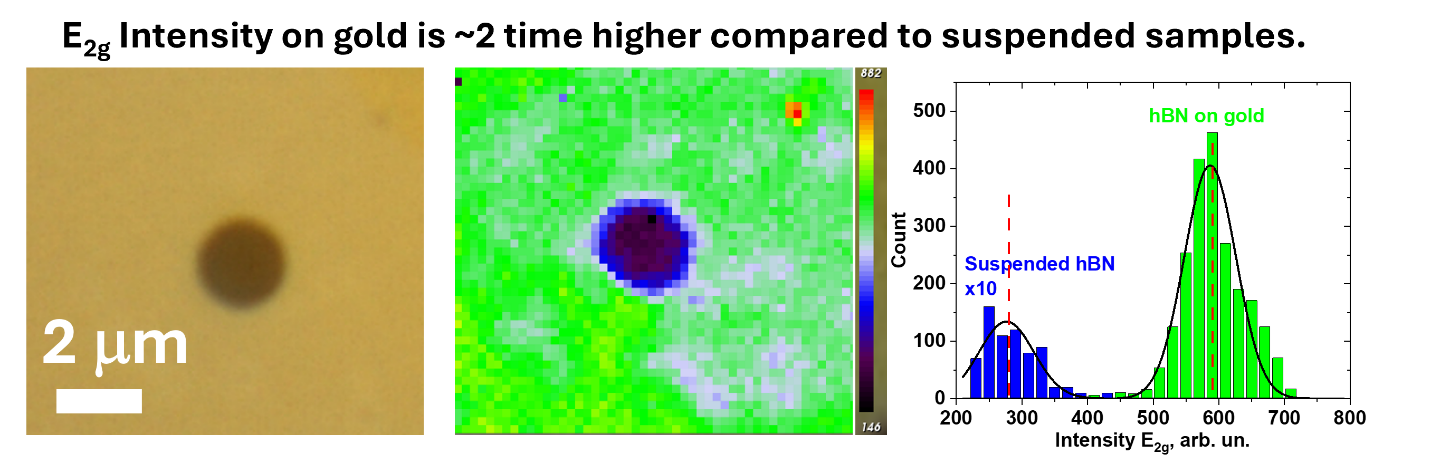


**Figure S7.** *Comparison of “as synthesized” hBN E_2g_ Raman band intensity in gold-supported and suspended regions.* Optical microscope image (left), E_2g_ Raman intensity map (center), and intensity histogram comparing suspended and supported areas. The E_2g_ intensity in regions supported by gold is approximately twice as high as in suspended regions (see Fig. S2 for reflectance estimations).


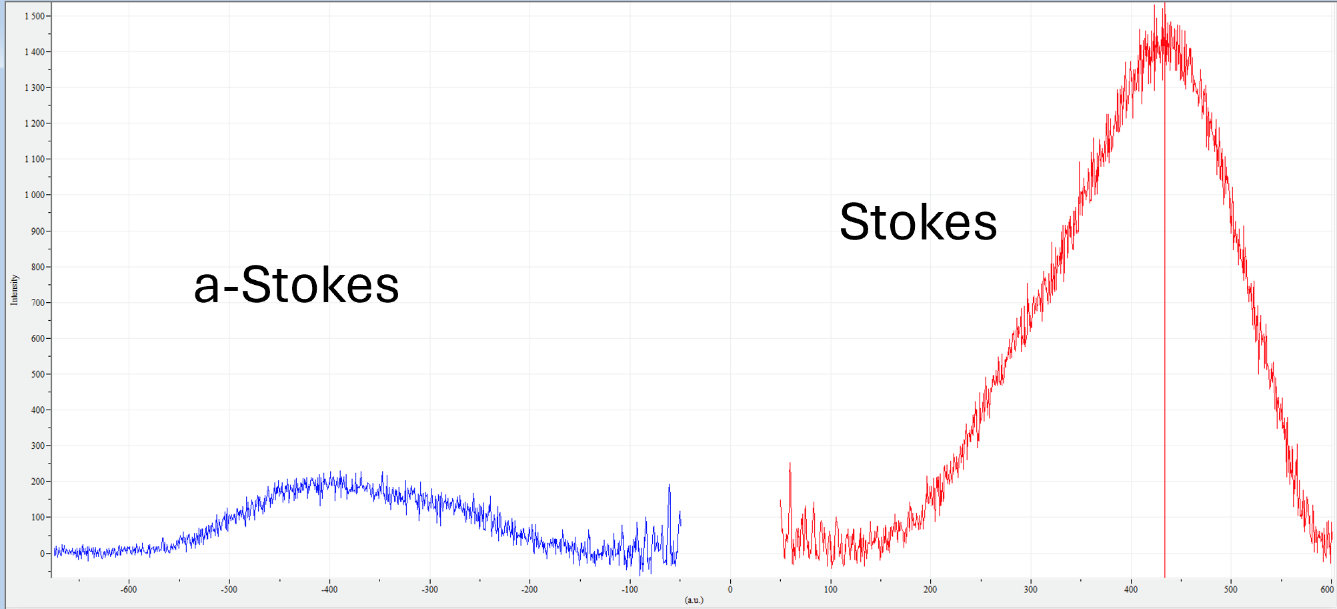


**Figure S8.** *Stokes and anti-Stokes shifts for the D band for 1.5 ions/nm² Ne⁺ bombardment of suspended hBN.* Excitation at 532 nm. The E₂_g_ and E’ modes have larger Raman shifts and, consequently, much weaker intensities.


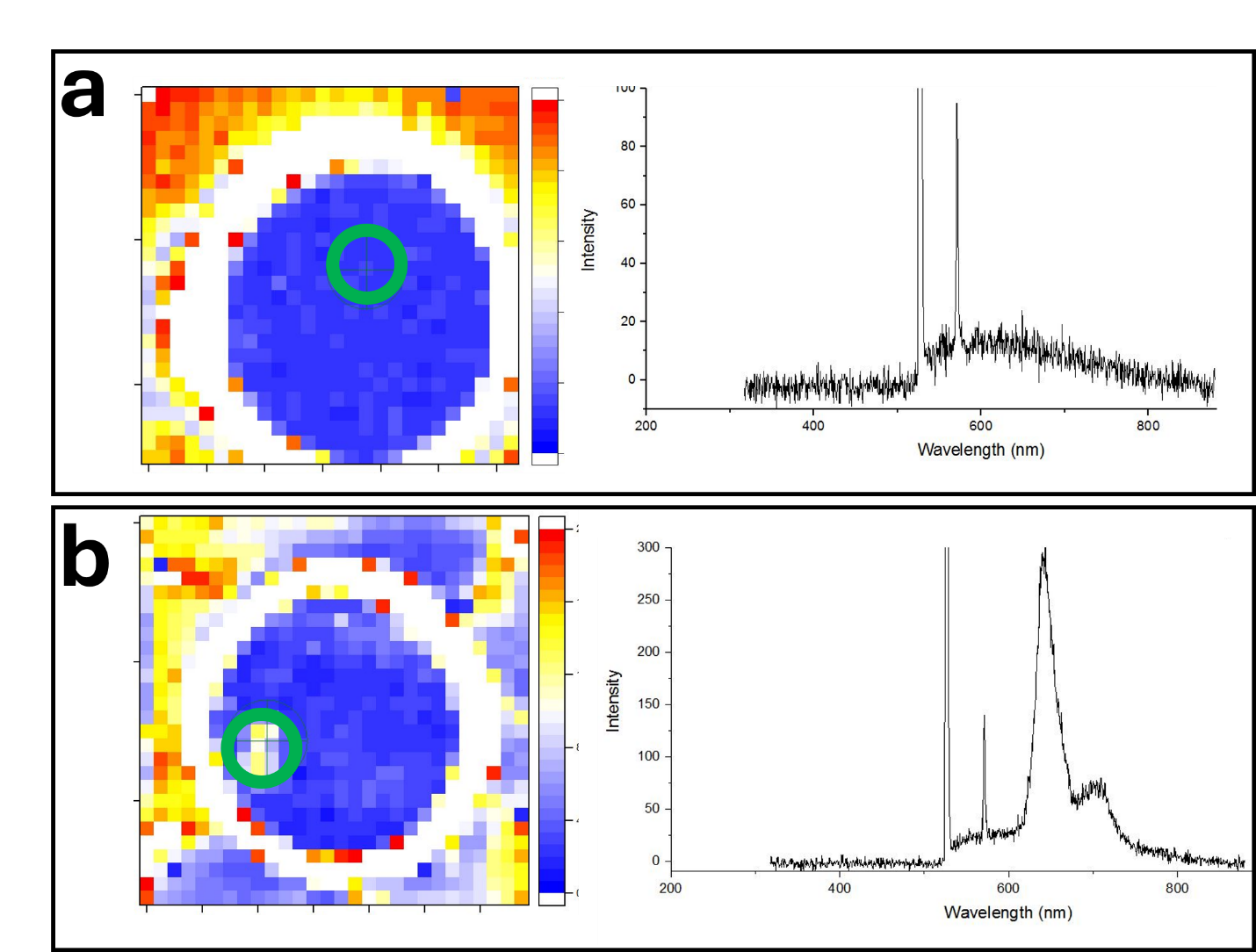


**Figure S9.** *PL maps of suspended “as-synthesized” hBN.* Most areas of the samples do not exhibit photoluminescence (PL), showing only sharp Raman E_2g_ signal (**a**). However, rare hotspots can be observed, which are attributed to single-photon emitter (SPE) centers (**b**). Such spots in our samples are very rare, their density is less than 10^-2^μm^-2^. ^2^

**
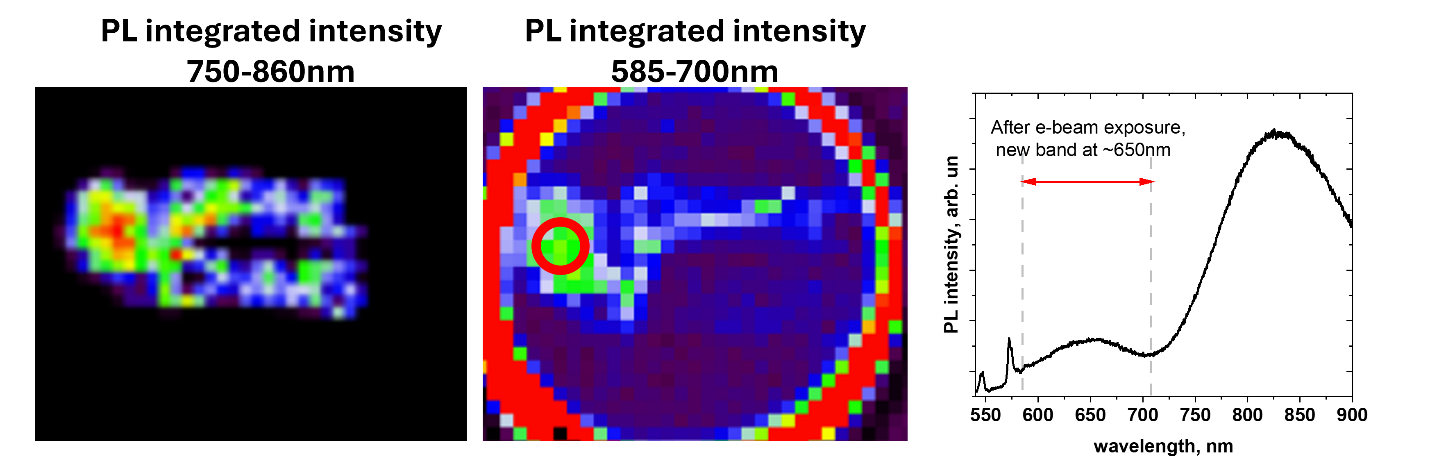
**

**Figure S10.** *PL maps of He^+^ bombarded samples taken after cathodoluminescence (CL) experiments.* The map shows the same region as in Fig. 1 of the main text, but at lower resolution. After CL experiment, the 800 nm band is still present (left), while a new 650 nm band emerges (center). The spectrum of the region highlighted by the red circle is shown on the right. It illustrates that additional defects of a different kind than V_B_ are formed during cathodoluminescence experiments.


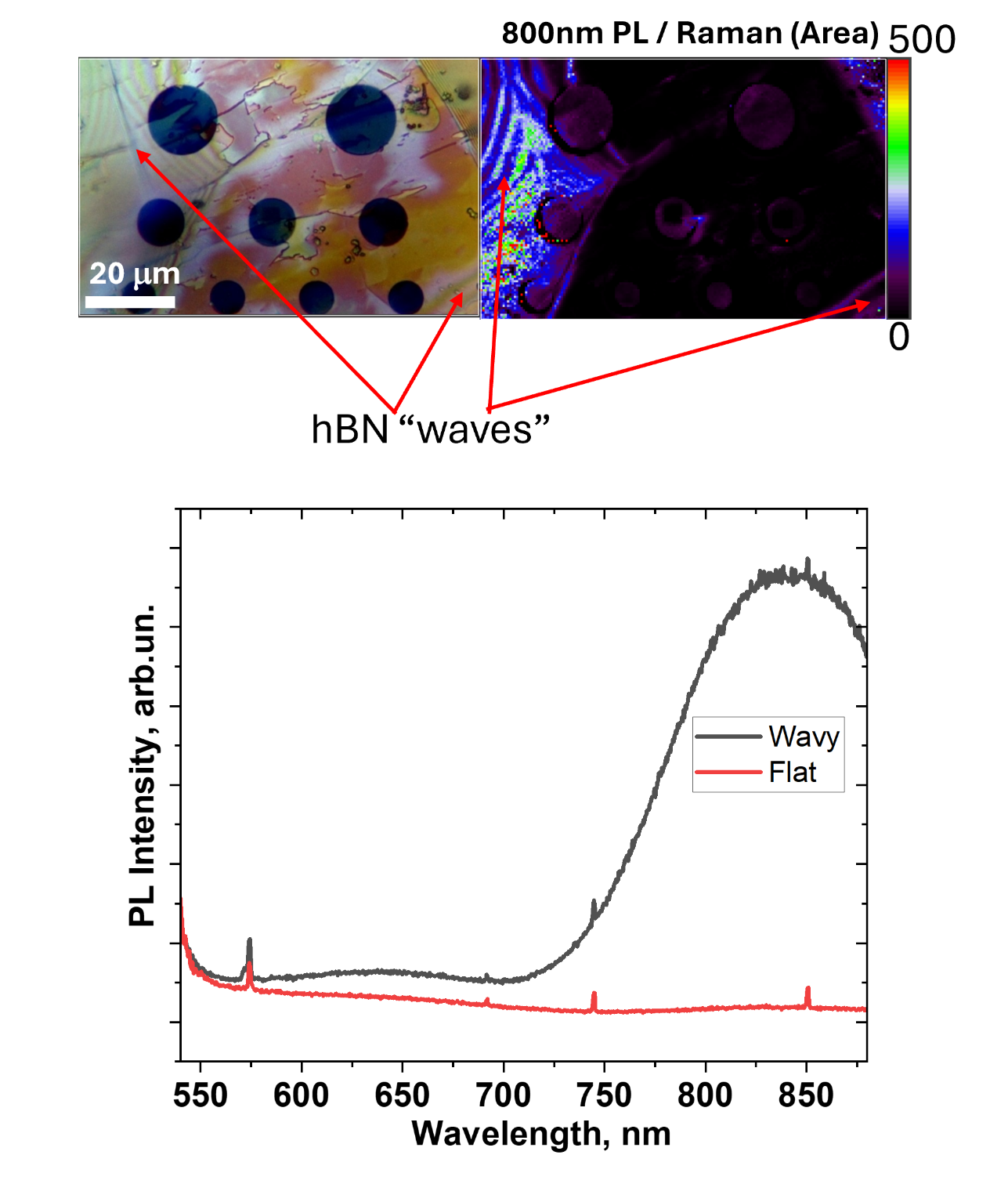


**Figure S11.** *Illustration of the intense 800 nm PL on “wavy” hBN***.** Optical microscope image of unintentional “wavy” hBN on a gold substrate; red arrows mark the wave locations (top, left). The integrated PL at 800 nm, normalized to the Raman signal (top, right). A comparison of the characteristic PL spectra recorded on a wavy feature on gold-supported versus suspended hBN (bottom). Note the minor 650 nm and E_2g_ bands. Bombardment was carried out at a low Ar^+^ ion dose (< 10^−2^ ions/nm²) by making a snapshot with the ion beam.

1. **Dependance of *ν_0,1,2_* on the He^+^ dosage**


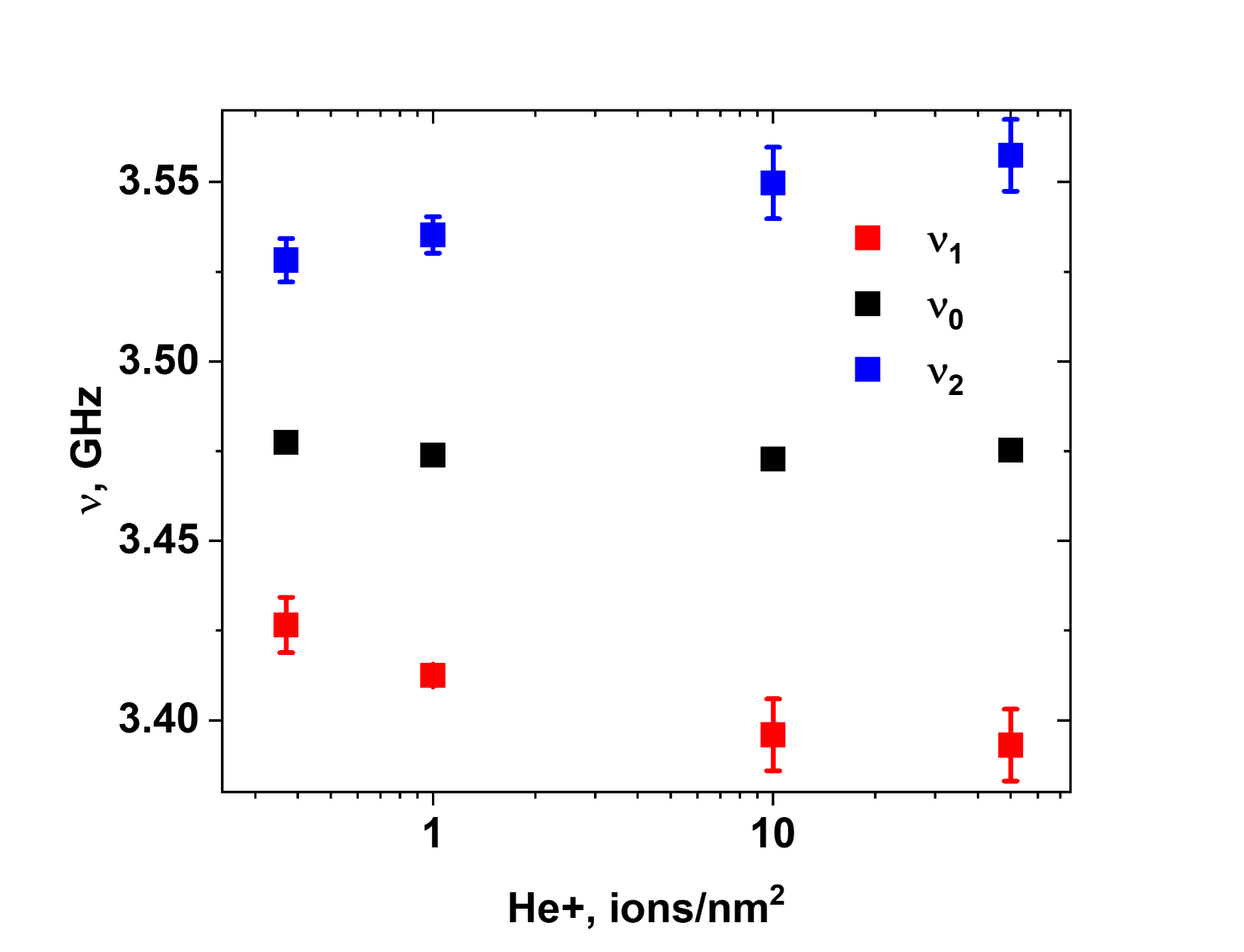


**Figure S12.** *Dependance of n_0,1,2_ on the He^+^ dosage.* ν_0_=D/h, while ν_2_-ν_1_=2E/h as described in the main text. Parameter *D* remains independent of the dosage, whereas the parameter *E* clearly exhibits a dosage-dependent trend.

1. **Neutrons bombardment.**

Bombardment by neutrons were done at High Flux Isotope Reactor at Oak Ridge National Laboratory^3^ Two samples were irradiated in the HFIR PT-1 transfer facility. A blank substrate was irradiated 20 s to check impurity levels and side-product activation. ^198^Au was the dominant gamma-ray emitting product. The hBN coated sample was irradiated also for 20 s for the impurity check and then irradiated 450 s the following day to dose the sample. All irradiations were performed in a graphite rabbit.

The longer irradiation fluence was calculated from activation results using an Au, Mn monitor pair. The Mn monitor determines the thermal neutron fluence rate and the Au mostly samples the epithermal one. The activation products, ^56^Mn and ^198^Au, are formed in dilute aluminum foils containing known monitor element concentrations.

After the sample irradiation, sufficient decay was allowed such that the sample was released without restriction for spectroscopic measurements.

**Table S1**. *PT-1 Fluences for three neutron energy ranges*

| **Energy** | **Fluence n/cm^2^** |
| --- | --- |
| **< 0.55 eV (Thermal)** | 2.07⋅10^17^ |
| **> 0.55 eV < 1 MeV (Epithermal)** | 6.26⋅10^16^ |
| **> 1 MeV (Fast)** | 6.44⋅10^15^ |

Thus, total neutron dose (all energies) was 2.8×10^17^ n/cm^2^ translating to total dose of ~2.8×10^3^ n/nm^2^. Sample was also simultaneously exposed to mean photon energy 1.3MeV with total dose of 7.8×10^14^ photons/cm^2^.

Since broad range of neutron energies were used, it is hard to decouple displacement or B-10 transmutation damage. For the latter, the main reaction to thermal neutrons is

^10^B +n 🡺 ^7^Li (0.8MeV) + ^4^He (1.5MeV) + γ (0.5MeV)

While for substrate (boron doped Si/SiN_x_/Ti/Au), neutron bombardment induces a wide range of reaction products leading to secondary hBN bombardment by species with diverse energy distributions, leading to additional defects in hBN. We expect substrate to experience only minor heating during irradiation, meaning that thermal effects – such as the conversion of V_B_ to N_B_V_N_ defects^4^ – should contribute negligibly.

**Figure S13.** *Neutron (top) and photon (bottom) energy spectra in High Flux Isotope Reactor.*

1. **Electrons bombardment.**

“As-synthesized” suspended hBN exposed to a 10 keV electron beam in a conventional SEM (~10^6^ e/nm^2^) exhibits a strong 650 nm PL accompanied by a pronounced ~1580 cm⁻¹ Raman band (**Fig. S14**). Both these bands may be attributed to the N_B_V_N_ - anti-site nitrogen vacancy in which a nitrogen atom migrates to a boron vacancy, leaving a neighboring nitrogen vacancy (Fig. 2b).^4^ Both phonon and PL spectra were calculated for N_B_V_N_,^5^ but lack of fingerprint features hinders unambiguous identification of this defect. It is not apparent why electrons are so prone in producing such defects. We suspect that it is due to a significant difference in the cross sections for producing initial defects and their ‘alteration’ by second collision, where the second one is significantly greater. It is apparent from **Fig. S14c** – there is no recognizable single atom knockout defects visible under electron irradiation, all defects appear with multiple atoms missing which simply grow in number and size with the dosage. Measurements of cross sections for inelastic scattering of electrons do show some indications for a preference of lower energy excitation. ^6, 7^

Possible electron-beam-induced carbon deposition,^8^ which also yields 1580 cm^-1^ Raman band complicates the analysis of keV e-beam bombardment results. The observed significant increase of 1580 cm^-1^ Raman band decreases in several days after exposure to ambient which may signify desorption of e-beam deposited hydrocarbons.


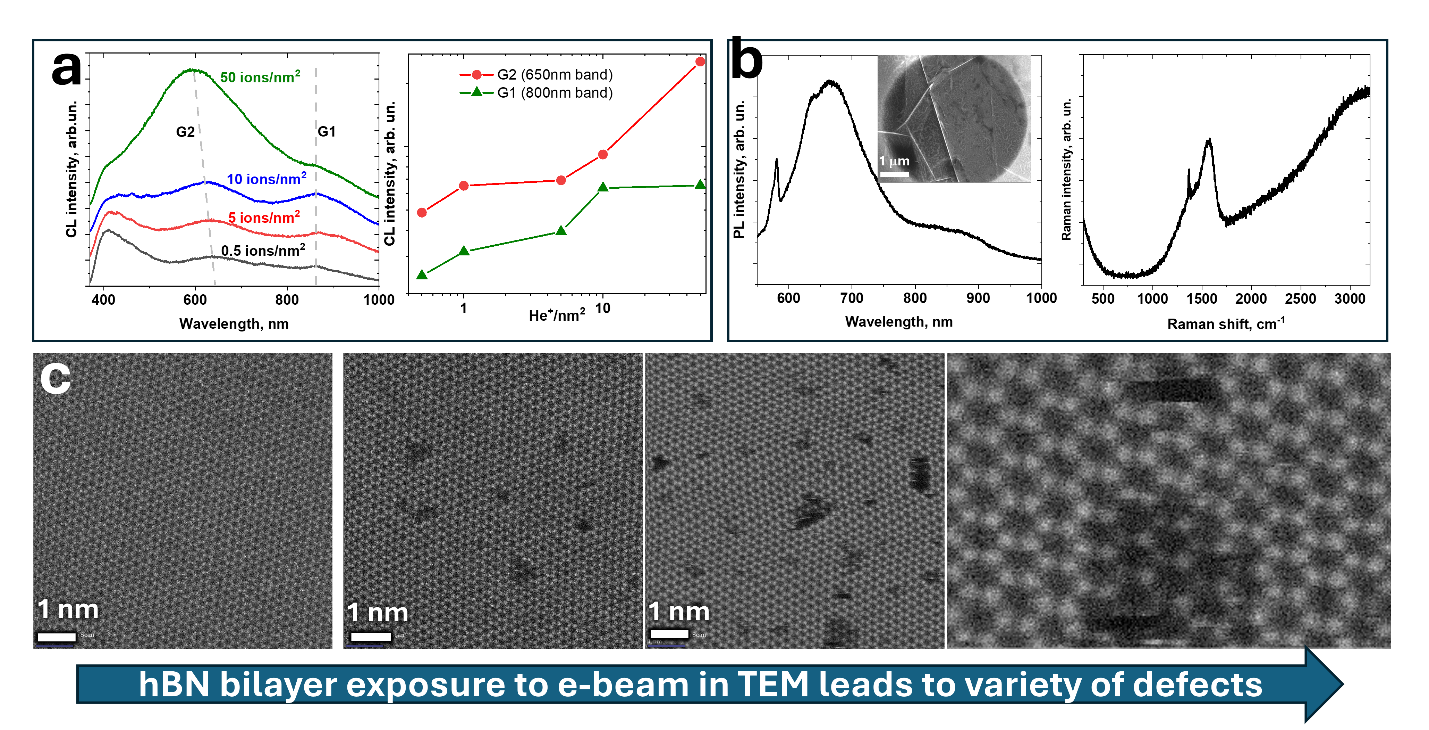


**Figure S14.** *Effects of electron beam irradiation on evolution of hBN defects.* **a.** CL spectra of suspended hBN bombarded with He^+^ (left) along with the dependence of 800 nm (G1) and 650 nm (G2) peak intensities on the He^+^ dosage (right). **b.** PL spectrum of “as-synthesized” suspended hBN after electron bombardment in a conventional SEM, accompanied by corresponding Raman spectrum (right). The inset shows an SEM image of the membrane. **c.** Formation and growth of defects in hBN observed during STEM imaging, *i.e.* e-beam bombardment. The three images correspond to ~0.4×10^8^, 1.5×10^8^ and 6.5×10^8^ e/nm^2^, respectively.

1. **End-of-Range (EOR) length estimations by Stopping Range of Ions in Matter (SRIM)**

Simulations were performed using Stopping Range of Ions in Matter (SRIM)^9^ using default conditions for substrate materials except for hBN density, which was manually corrected to 2.1 g/cm^3^ per Gautam et al.^10^ Simulations of 1,000 ions species (He^+^, Ne^+^, and Ar^+^) at 30 keV were performed on hBN thicknesses of 90 and 250 nm on a 250 nm film of Au (depth scales of plots are fixed at 325 nm for simple comparison). In all cases monolayer collision steps were chosen for improved accuracy. It was found that significant interaction with the underlying gold film occurs for He^+^ with both hBN thicknesses (**Fig S15a,b**) and Ne^+^ on the 90 nm hBN film (**Fig S15d**). The high mass of Ar^+^ causes stopping of all ions well within the hBN film in both the 250 nm and 90 nm thicknesses (**Fig S15c,d**, respectively). Ne^+^ remains confined in the 250 nm hBN film (**Fig S15c**) but slightly penetrates into the gold film when the thickness of hBN is reduced to 90 nm (**Fig S15d**). It should be noted that the cases where significant penetration into the high mass Au film occurs, the potential for backscattering of ions back into the hBN increases and the likelihood of ions stopping near the bottom of the hBN at the Au interface increases significantly, which may lead to a higher defect concentrations.


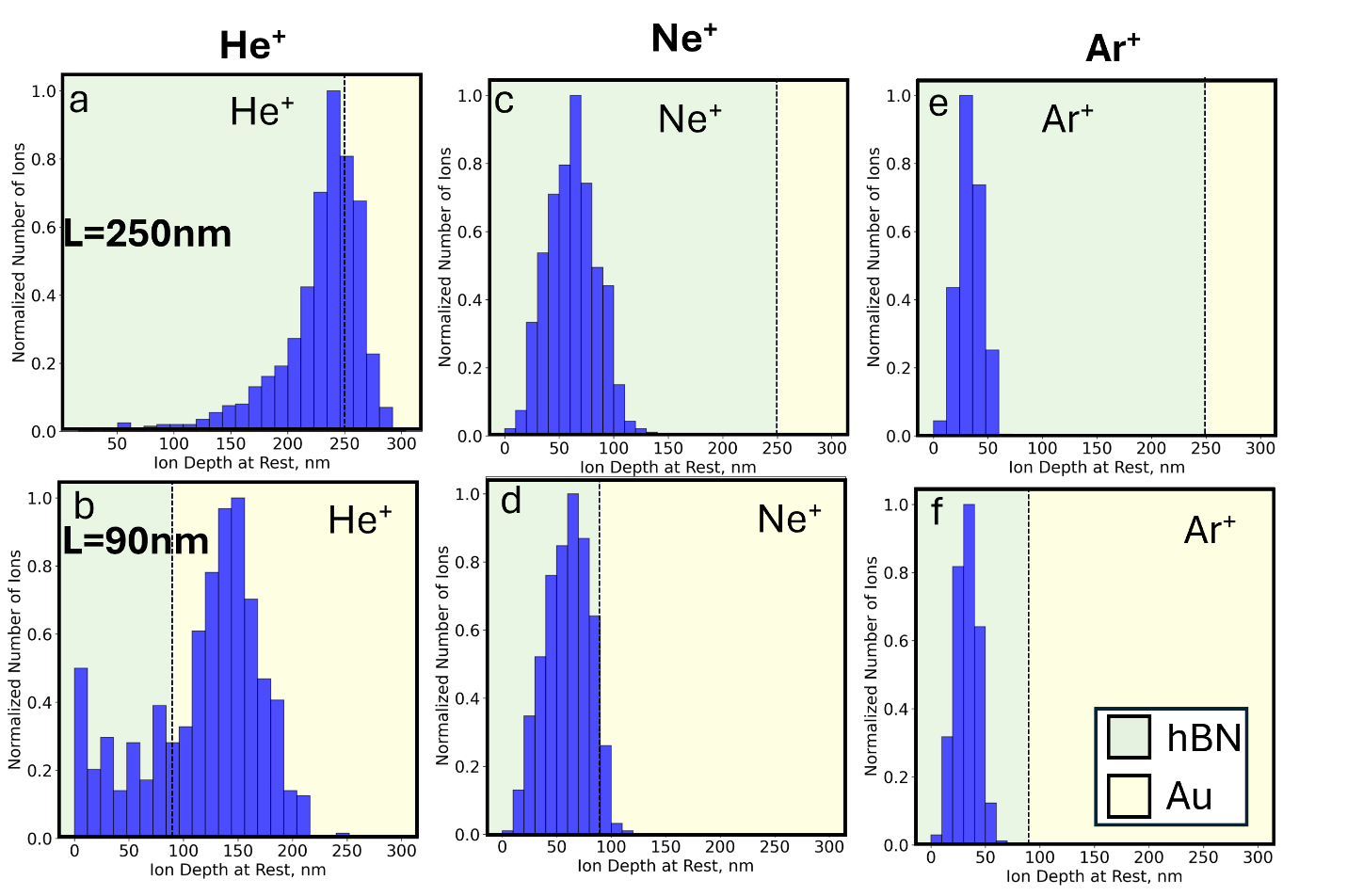


**Figure S15.** *Simulated, normalized ion penetration depth distributions as a function of ion species and substrate materials.* **a.** 30 keV He^+^ on 250nm hBN/250nm Au. **b.** 30 keV He^+^ on 90nm hBN/250nm Au. **c.** 30 keV Ne^+^ on 250nm hBN/250nm Au. **d.** 30 keV Ne^+^ on 90nm hBN/250nm Au. **e.** 30 keV Ar^+^ on 250nm hBN/250nm Au. **e.** 30 keV Ar^+^ on 90nm hBN/250nm Au.

1. **XPS spectra of hBN after He^+^ bombardment.**

*
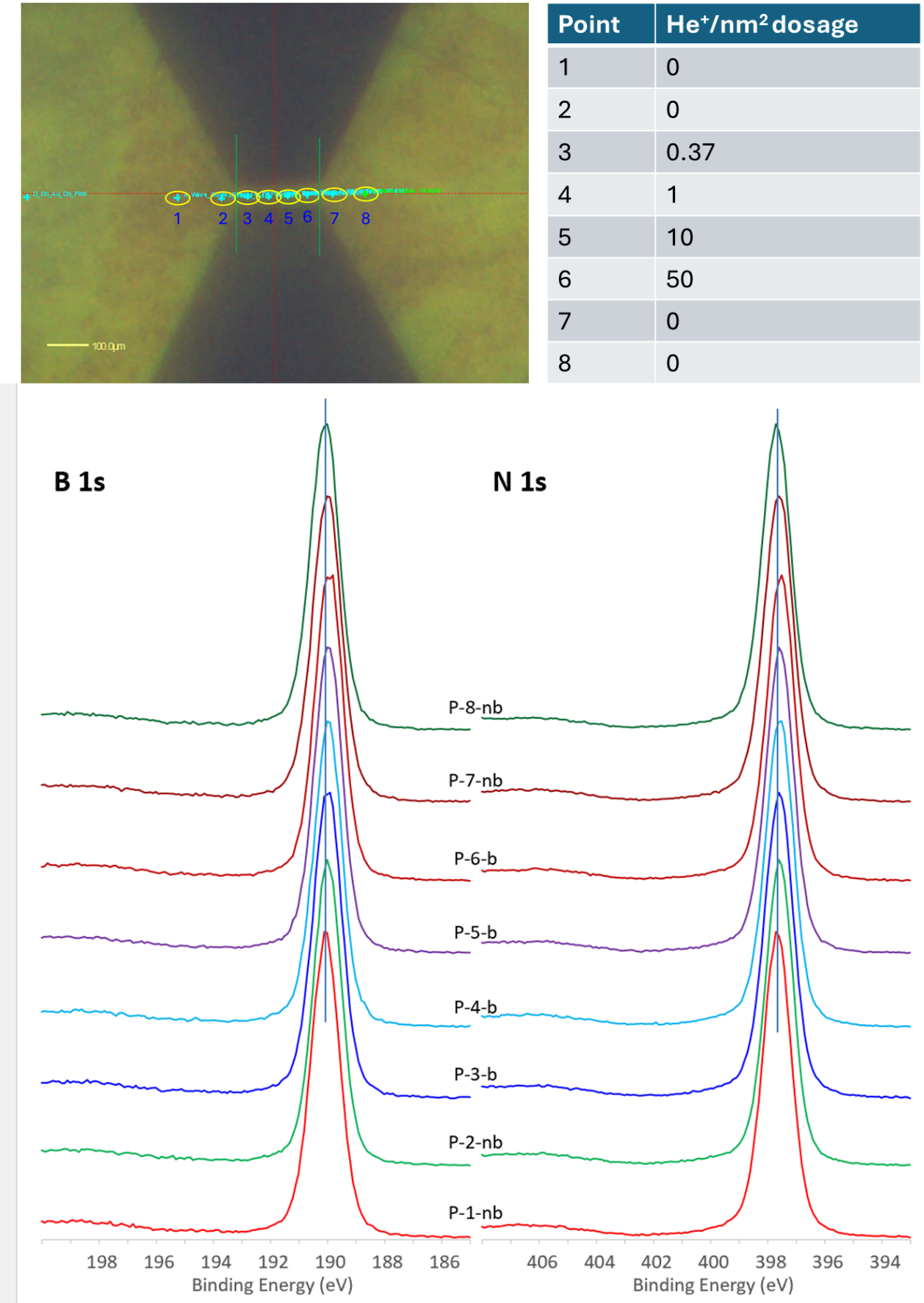
*

**Figure S16.** *XPS spectra of hBN after He⁺ bombardment*. The optical image of hBN on the waveguide highlighted points corresponding to different He⁺ doses, as listed in the table on the right. XPS spectra of bombarded hBN up to 50 He⁺/nm² are shown with the respective doses. The spectra of bombarded and unbombarded samples coincide at least up to the dose of 50 He⁺/nm²

1. **ODMR at 650nm.**


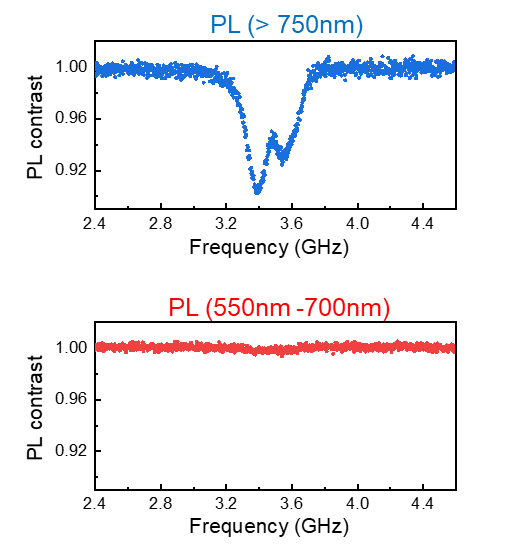


**Figure S17.** *ODMR at 650 nm.* The 650 nm PL does not exhibit an ODMR signal, at least within 2.4–5 GHz and at zero field.

1. **References.**

[1] Sarkar, S.; Xu, Y.; Mathew, S.; Lal, M.; Chung, J. Y.; Lee, H. Y.; Watanabe, K.; Taniguchi, T.; Venkatesan, T.; Gradecak, S. Identifying Luminescent Boron Vacancies in h-BN Generated Using Controlled He Ion Irradiation. *Nano Lett* **2023**, *24* (1), 43-50.

[2] Vlassiouk, I.; Smirnov, S.; Puretzky, A.; Olunloyo, O.; Geohegan, D. B.; Dyck, O.; Lupini, A. R.; Unocic, R. R.; Meyer, H. I. I. I.; Xiao, K.; et al. Armor for Steel: Facile Synthesis of Hexagonal Boron Nitride Films on Various Substrates (Adv. Mater. Interfaces 1/2024). *Adv Mater Interfaces* **2024**, *11* (1).

[3] *High Flux Isotope Reactor*. https://neutrons.ornl.gov/hfir.

[4] Venturi, G.; Chiodini, S.; Melchioni, N.; Janzen, E.; Edgar, J. H.; Ronning, C.; Ambrosio, A. Selective Generation of Luminescent Defects in Hexagonal Boron Nitride. *Laser Photonics Rev* **2024**, *18* (6).

[5] Tawfik, S. A.; Ali, S.; Fronzi, M.; Kianinia, M.; Tran, T. T.; Stampfl, C.; Aharonovich, I.; Toth, M.; Ford, M. J. First-principles investigation of quantum emission from hBN defects. *Nanoscale* **2017**, *9* (36), 13575-13582.

[6] de Vera, P.; Garcia-Molina, R. Electron Inelastic Mean Free Paths in Condensed Matter Down to a Few Electronvolts. *J Phys Chem C* **2019**, *123* (4), 2075-2083.

[7] Tanuma, S.; Shiratori, T.; Kimura, T.; Goto, K.; Ichimura, S.; Powell, C. J. Experimental determination of electron inelastic mean free paths in 13 elemental solids in the 50 to 5000 eV energy range by elastic-peak electron spectroscopy. *Surf Interface Anal* **2005**, *37* (11), 833-845.

[8] Lau, D.; Hughes, A. E.; Muster, T. H.; Davis, T. J.; Glenn, A. M. Electron-Beam-Induced Carbon Contamination on Silicon: Characterization Using Raman Spectroscopy and Atomic Force Microscopy. *Microsc Microanal* **2010**, *16* (1), 13-20.

[9] Ziegler, J. F. Srim-2003. *Nucl Instrum Meth B* **2004**, *219*, 1027-1036.

[10] Gautam, C.; Tiwary, C. S.; Lose, S.; Brunetto, G.; Ozden, S.; Vinod, S.; Raghavan, P.; Biradar, S.; Galvao, D. S.; Ajayan, P. M. Synthesis of Low-Density, Carbon-Doped, Porous Hexagonal Boron Nitride Solids. *Acs Nano* **2015**, *9* (12), 12088-12095.
